# Physical activity is associated with slower epigenetic ageing—Findings from the Rhineland study

**DOI:** 10.1111/acel.13828

**Published:** 2023-04-10

**Authors:** Fabienne A. U. Fox, Dan Liu, Monique M. B. Breteler, Nasir Ahmad Aziz

**Affiliations:** ^1^ Population Health Sciences, German Center for Neurodegenerative Diseases (DZNE) Bonn Germany; ^2^ Institute for Medical Biometry, Informatics and Epidemiology (IMBIE), Faculty of Medicine University of Bonn Bonn Germany; ^3^ Department of Neurology, Faculty of Medicine University of Bonn Bonn Germany

**Keywords:** biological age, cardiovascular diseases, cardiovascular risk factors, cohort studies, DNA methylation, epidemiology, epigenetics, exercise, immune function, public health

## Abstract

Epigenetic ageing, i.e., age‐associated changes in DNA methylation patterns, is a sensitive marker of biological ageing, a major determinant of morbidity and functional decline. We examined the association of physical activity with epigenetic ageing and the role of immune function and cardiovascular risk factors in mediating this relation. Moreover, we aimed to identify novel molecular processes underlying the association between physical activity and epigenetic ageing. We analysed cross‐sectional data from 3567 eligible participants (mean age: 55.5 years, range: 30–94 years, 54.8% women) of the Rhineland Study, a community‐based cohort study in Bonn, Germany. Physical activity components (metabolic equivalent (MET)‐Hours, step counts, sedentary, light‐intensity and moderate‐to‐vigorous intensity activities) were recorded with accelerometers. DNA methylation was measured with the Illumina HumanMethylationEPIC BeadChip. Epigenetic age acceleration (Hannum's age, Horvath's age, PhenoAge and GrimAge) was calculated based on published algorithms. The relation between physical activity and epigenetic ageing was examined with multivariable regression, while structural equation modeling was used for mediation analysis. Moreover, we conducted an epigenome‐wide association study of physical activity across 850,000 CpG sites. After adjustment for age, sex, season, education, smoking, cell proportions and batch effects, physical activity (step counts, MET‐Hours and %time spend in moderate‐to‐vigorous activities) was non‐linearly associated with slower epigenetic ageing, in part through its beneficial effects on immune function and cardiovascular health. Additionally, we identified 12 and 7 CpGs associated with MET‐Hours and %time spent in moderate‐to‐vigorous activities, respectively (*p* < 1 × 10^−5^). Our findings suggest that regular physical activity slows epigenetic ageing by counteracting immunosenescence and lowering cardiovascular risk.

## Graphical abstract

In this study, we examined the relation between physical activity and epigenetic ageing in 3567 eligible participants of a large community‐based, prospective cohort study, the Rhineland Study. We show that physical activity is associated with slower epigenetic ageing (GrimAge acceleration and PhenoAge acceleration), which was in part mediated by cardiovascular health and immune function.

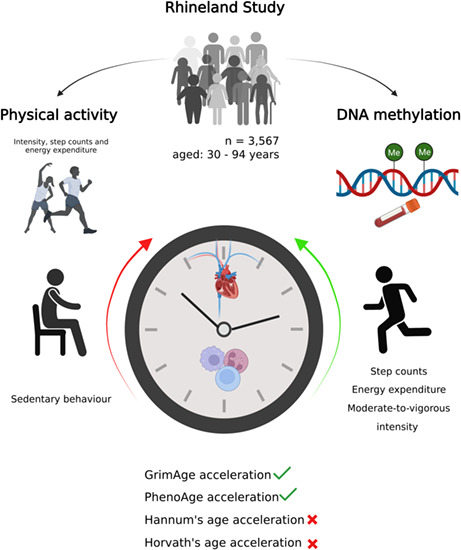

**Abbreviations**
ASCVD Risk Score: assessment of cardiovascular disease risk score
Bas: basophils
B memory: B memory cells
BMI: body mass index
B naïve: naïve B cells
BP: biological process
CC: cellular component
CD4 memory: memory CD4T cells
CD8 naïve: naïve CD8T cells
CD8 memory: memory CD8T cells
Chr: chromosome
(95%) CI: confidence interval
CpG: cytosine‐phosphate‐guanine
DBP: diastolic blood pressure
DNA: deoxyribonucleic acid
Eos: eosinophils
ESC SCORE2: European Society of Cardiology Score
EWAS: epigenome‐wide association analysis
FAMD: factor analysis for mixed data
GO: Gene Ontology
GWAS: genome‐wide association studies
HDL: high‐density lipoprotein
LDL: low‐density lipoprotein
MET‐Hours: metabolic equivalent hours
MF: molecular function
Mono: monocytes
mQTL: methylation quantitative trait loci
MVPA: moderate‐to‐vigorous physical activity
Neu: neutrophils
NK: natural killer cells
PCA: principle component analysis
Pos: position
ref: reference group
SEM: structural equation modelling
SBP: systolic blood pressure
SD: standard deviation
Treg: regulatory T cells
WHR: waist‐to‐hip ratio

## 1 INTRODUCTION

Physical activity has been associated with a decreased risk of age‐associated diseases, an increased life expectancy, and a higher quality of life (World Health Organization, 2020). Several physiological, biochemical, and transcriptional changes have been observed in response to acute exercise as well as regular physical activity, which may underlie these benefits (Neufer et al., 2015). Specifically, exercise has been observed to affect the methylation status of genes, with some genes showing hypomethylation and others hypermethylation in response to exercise (Jacques et al., 2019; Voisin et al., 2015).

Dynamic DNA methylation regulates gene expression and is responsive to environmental and lifestyle changes (Moore et al., 2013). DNA methylation at specific Cytosine‐phosphate‐Guanine (CpG) sites has been linked to age‐associated functional decline and has been suggested as a signature of biological ageing as estimated through *epigenetic clocks* (Hannum et al., 2013). By now, several epigenetic clocks have been developed (Hannum et al., 2013; Horvath, 2013; Levine et al., 2018; Lu et al., 2019). Whereas first‐generation clocks (i.e., Hannum and Horvath's clocks) focus on predicting chronological age, second‐generation clocks (i.e., GrimAge and PhenoAge) were developed using clinically relevant biomarkers and a range of different proteins to reflect mortality risk (Topart et al., 2020). The second‐generation clocks are therefore also referred to as lifespan estimators. The difference between an individual's estimated biological age and chronological age is referred to as epigenetic ageing, including HorvathAge acceleration, HannumAge acceleration, PhenoAge acceleration, and GrimAge acceleration (Jain et al., 2022; Kim et al., 2021; McCrory et al., 2021; O'Shea et al., 2022).

Physical activity may protect against age‐associated functional decline and may slow epigenetic ageing (Oblak et al., 2021). Specifically, higher levels of self‐reported physical activity have been associated with a lower GrimAge (Kresovich et al., 2021) and Hannum age (Quach et al., 2017). However, large‐scale studies assessing the relation between physical activity and epigenetic ageing are sparse. A systematic review reported physical activity to be associated with slower biological ageing as reflected by second‐generation clocks, including PhenoAge and GrimAge, but not when using first‐generation clocks (Oblak et al., 2021). Importantly, these previous studies predominantly used questionnaire‐based physical activity assessments, which are susceptible to overreporting and cannot discriminate among the different physical activity components (Chastin et al., 2018). To the best of our knowledge, only one study to date has examined the association of objective accelerometer‐based physical activity and epigenetic ageing, though only in older adults (Gale et al., 2018). This study found that higher step counts were related to a lower Hannum's age, whereas more sit‐to‐stand transitions were related to a higher Horvath's age in 79‐year old adults.

Thus far, the mechanisms through which physical activity affects age‐associated functional decline are poorly understood. A recent study observed a link between the proportion of naïve and activated T and NK cells and DNA methylation, suggesting that epigenetic ageing may be driven by immunosenescence (Jonkman et al., 2022). Physical activity has been found to directly affect lymphocyte ß2‐adrenergic receptor sensitivity, leading to an increased mobilization of T and NK cells, immune surveillance and progenitor cell mobilization, which in turn causes less viral burden on the T cell compartment and reduces the accumulation of senescent T cells (Duggal et al., 2019). Similarly, regular physical activity leads to a reduced cardiovascular disease risk (World Health Organization, 2020). Poor cardiovascular health increases the risk of age‐associated functional decline and has been related to a faster GrimAge acceleration (Joyce et al., 2021). It is unclear, however, whether the advantageous effects of physical activity on epigenetic ageing are solely mediated through their effects on immune function and cardiovascular health or through other (partially) independent mechanisms.

We therefore aimed to examine whether distinct, objectively assessed physical activity components are associated with slower biological ageing in adults over a wide age range. To this end, we leveraged cross‐sectional data of a large community‐based cohort‐study and assessed whether accelerometer‐derived physical activity is associated with epigenetic age acceleration. We focused on the effects of physical activity components on GrimAge acceleration because (1) second‐generation epigenetic clocks have been demonstrated to more closely reflect the high inter‐individual variability in the underlying biological ageing processes as compared to first‐generation epigenetic clocks, (2) GrimAge acceleration has been demonstrated to be the strongest predictor of age‐associated functional decline (Lu et al., 2019; McCrory et al., 2021), and (3) GrimAge acceleration has been found to outperform the other epigenetic clocks, both in predicting mortality risk (Lu et al., 2019; McCrory et al., 2021) and in capturing multisystem dysregulation (Liu et al., 2023). Nevertheless, for comparison, we also explored the association between physical activity and Hannum's age, Horvath's age and PhenoAge acceleration in additional sensitivity analyses. Furthermore, we investigated to what extent the effect of physical activity on GrimAge acceleration is mediated through its effects on immune function and cardiovascular risk factors, while also examining potential reversed mediation effects—whether GrimAge acceleration mediates the association between physical activity and markers of cardiovascular health. Lastly, we performed an epigenome‐wide association study of physical activity and conducted a gene enrichment analysis to gain biological insights into the mechanisms underlying the effects of physical activity on epigenetic ageing.

## 2 RESULTS

### 2.1 Sample characteristics

The sample characteristics are presented in Tables 1 and S1. In our main analysis, 3567 eligible participants were included, of whom 1955 were women (54.8%). Participants' mean age was 55.5 years (*SD*: 14.1, age range: 30–94 years). Participants had on average high education and physical activity levels. Physical activity levels were lower in older adults compared to younger adults (Table S1).

**TABLE 1:** Sample demographics.

	Eligible participants (*n* = 3567)	Individuals with cardiovascular data (*n* = 3357)	Excluded participants (*n* = 1429)	*p* ^a^
Age (years), mean (*SD*)	55.5 (14.1)	55.4 (14.0)	54.2 (13.7)	0.002
30–39	594 (16.7)	563 (16.8)	238 (16.7)	[ref]
40–49	610 (17.1)	575 (17.1)	315 (22.0)	0.019
50–59	966 (27.1)	914 (27.2)	392 (27.4)	0.986
60–69	747 (20.9)	707 (21.1)	261 (18.3)	0.172
70–79	492 (13.8)	463 (13.8)	173 (12.1)	0.273
80–89	154 (4.3)	132 (3.9)	45 (3.2)	0.096
90+	4 (0.1)	3 (0.1)	5 (0.4)	0.093
Sex (women), *n* (%)	1955 (54.8)	1821 (54.2)	865 (60.5)	<0.001
Body‐mass index (kg/m^2^), mean (*SD*)	25.86 (4.38)	25.86 (4.33)	26.12 (4.95)	0.008
Waist‐to‐hip ratio, mean (*SD*)	0.87 (0.10)	0.87 (0.10)	0.86 (0.10)	0.205
Cardiovascular event, *n* (% Yes)	319 (8.9)	289 (8.6)	102 (7.1)	0.338
Smoking, *n* (% Yes)	448 (12.6)	420 (12.5)	173 (12.2)	0.658
Diabetes, *n* (% Yes)	192 (5.4)	179 (5.3)	69 (4.8)	0.993
Hypertension, *n* (%)				
No	2171 (61.5)	2086 (62.1)	893 (63.4)	[ref]
Yes, controlled	613 (17.4)	575 (17.1)	245 (17.4)	0.393
Yes, uncontrolled	699 (19.8)	664 (19.8)	268 (19.0)	0.582
Yes, unknown	46 (1.3)	32 (1.0)	3 (0.2)	0.557
Education ISCED11, *n* (%)				
High	1867 (52.3)	1751 (52.2)	751 (54.3)	[ref]
Middle	1629 (45.7)	1542 (45.9)	602 (43.5)	0.249
Low	71 (2.0)	64 (1.9)	30 (2.2)	0.709
Actimetry season, *n* (%)				
Spring	752 (21.1)	677 (20.17)	328 (23.85)	[ref]
Summer	823 (23.1)	745 (22.19)	363 (26.40)	0.877
Autumn	1016 (28.5)	984 (29.31)	410 (29.82)	0.454
Winter	976 (27.4)	951 (28.33)	274 (19.93)	<0.001
Daily Sensor Hours Worn (hours), mean (*SD*)	23.81 (0.51)	23.81 (0.52)	23.74 (0.76)	0.039
Daily Energy Expenditure (MET‐Hours), mean (*SD*)	33.98 (1.37)	33.99 (1.37)	33.25 (2.05)	<0.001
Daily Step Count, mean (*SD*)	8804.97 (3235.32)	8834.85 (3235.77)	7826.58 (3960.50)	<0.001
% Daily Light Intensity Physical Activity, mean (*SD*)	21.26 (6.08)	21.27 (6.08)	20.86 (9.21)	0.029
% Daily Moderate‐to‐Vigorous Physical Activity, mean (*SD*)	4.70 (1.79)	4.72 (1.79)	4.58 (1.97)	0.455
% Daily Sedentary, mean (*SD*)	74.03 (6.72)	74.01 (6.71)	76.66 (9.43)	<0.001
Hannum's Age acceleration, mean (*SD*)	0.31 (5.67)	0.11 (5.60)	0.20 (5.58)	0.840
Horvath's Age acceleration, mean (*SD*)	0.22 (5.28)	0.09 (5.24)	0.40 (5.23)	0.208
PhenoAge acceleration, mean (*SD*)	0.17 (6.61)	0.04 (6.60)	0.18 (6.57)	0.837
GrimAge acceleration, mean (*SD*)	0.01 (7.41)	−0.11 (7.42)	−0.34 (7.48)	0.533
% Basophils, mean (*SD*)^b^	0.77 (0.81)	0.78 (0.81)	0.78 (0.78)	0.674
% Eosophils, mean (*SD*) ^b^	2.44 (1.82)	2.46 (1.81)	2.48 (1.90)	0.668
% Neutrophils, mean (*SD*)^b^	50.14 (11.66)	49.96 (11.67)	50.34 (11.51)	0.186
% Monophils, mean (*SD*)^b^	8.95 (2.20)	8.97 (2.20)	8.79 (2.20)	0.076
% Naïve B cells, mean (*SD*)^b^	3.02 (1.74)	3.06 (1.74)	2.99 (1.58)	0.204
% Memory B cells, mean (*SD*)^b^	2.05 (3.16)	2.07 (3.24)	1.92 (1.45)	0.219
% Naïve CD4 T cells, mean (*SD*)^b^	6.76 (4.32)	6.83 (4.32)	6.92 (4.15)	0.576
% Memory CD4 T cells, mean (*SD*)^b^	11.15 (4.00)	11.14 (3.98)	11.16 (4.01)	0.913
% Regulatory T cells, mean (*SD*)^b^	0.08 (0.32)	0.08 (0.32)	0.09 (0.33)	0.571
% Naïve CD8 T cells, mean (*SD*)^b^	2.25 (2.08)	2.29 (2.08)	2.36 (2.15)	0.608
% Memory CD8 T cells, mean (*SD*)^b^	6.84 (5.69)	6.78 (5.66)	6.70 (5.28)	0.540
% Natural killer cells, mean (*SD*)^b^	5.67 (2.58)	5.70 (2.59)	5.67 (2.61)	0.811
Systolic blood pressure (mm Hg), mean (*SD*)	126.60 (16.04)	126.68 (16.00)	125.99 (16.06)	0.358
Diastolic blood pressure (mm Hg), mean (*SD*)	75.34 (9.47)	75.40 (9.44)	75.40 (9.24)	0.231
Cholesterol (mg/dL), mean (*SD*)	198.28 (39.74)	198.33 (39.68)	199.10 (37.39)	0.297
High‐density lipoprotein (mg/dL), mean (*SD*)	62.32 (17.74)	62.29 (17.73)	63.00 (17.64)	0.951
Low‐density lipoprotein (mg/dL), mean (*SD*)	126.49 (36.17)	126.58 (36.15)	126.35 (34.05)	0.625
Triglycerides (mg/dL), mean (*SD*)	111.13 (69.35)	111.07 (69.46)	111.68 (67.25)	0.188
Insulin (mU/L), mean (*SD*)	10.43 (7.51)	10.40 (7.50)	10.06 (8.11)	0.370

Abbreviations: MET‐Hours, Metabolic‐Equivalent Hours; ref, reference group; *SD*, standard deviation; 95% CI; 95% confidence interval.

^a^ Group differences (included vs. excluded participants) were assessed using binomial logistic regression, adjusted for age and sex (group differences for the variables age and sex were only adjusted for the other respectively).

^b^ Leukocyte subtypes were derived based on DNA methylation levels (as described by Salas et al. 2022).

### 2.2 Effects of physical activity on epigenetic age acceleration

**FIGURE 1:**
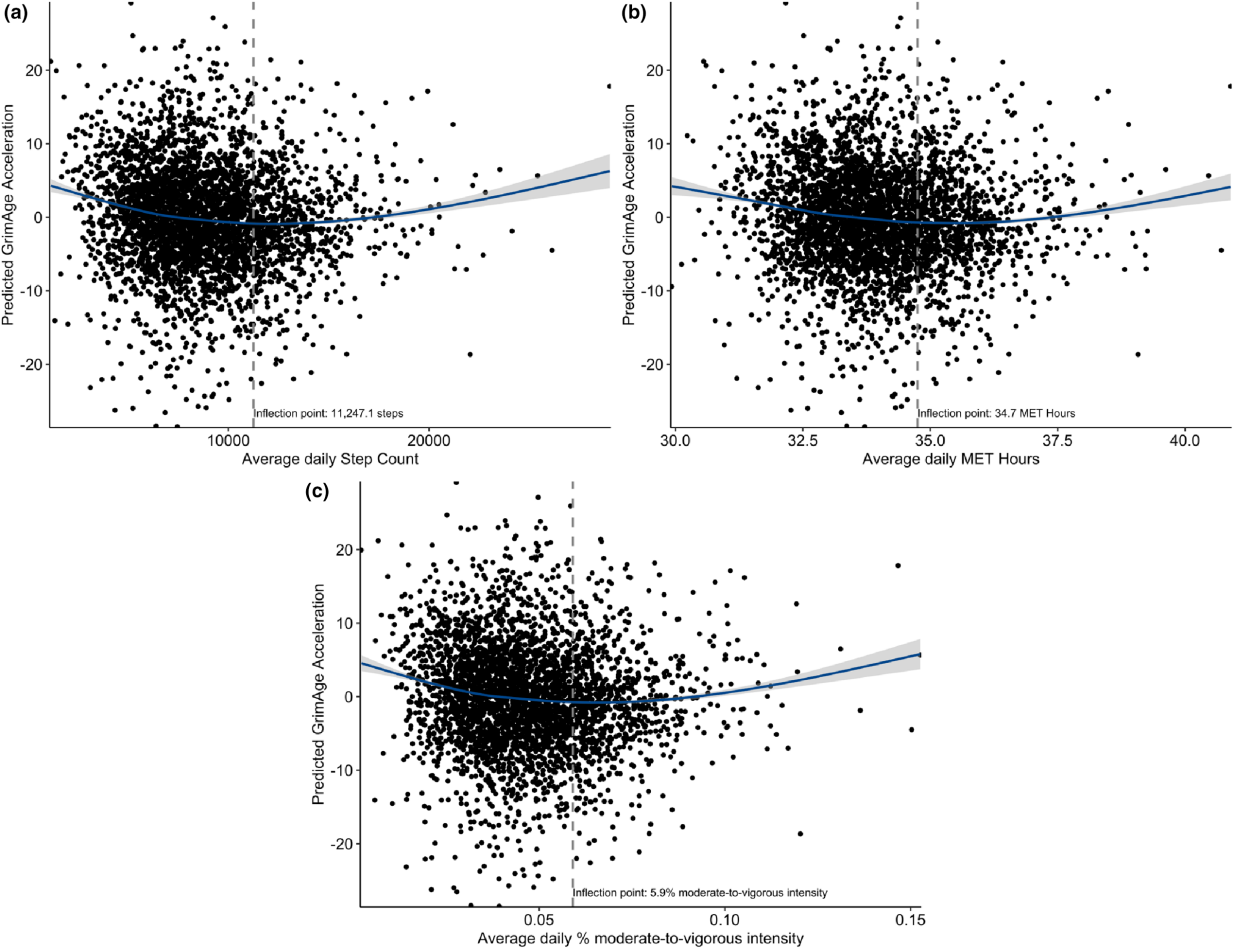
Scatterplot of association between physical activity and GrimAge acceleration. Regression lines were adjusted for age, age^2^, sex, education, batch effect, cell proportions, season and smoking status. MET‐Hours, Metabolic‐Equivalent Hours.

Using polynomial regression models, we examined the effects of physical activity on epigenetic ageing. Higher average daily step counts and energy expenditure, as measured in metabolic equivalent (MET‐) Hours, were non‐linearly associated with lower GrimAge acceleration (Table 2a). For example, for step counts, the difference in GrimAge acceleration for an individual with −2 standard deviations below the mean compared to an individual with an average daily step count (corresponding to ~2300 and ~ 8800 steps a day, respectively) was around 21 months. Similarly, for MET‐Hours, the difference in GrimAge acceleration for an individual with −2 standard deviations below the mean compared to one with an average amount of MET‐Hours (corresponding to ~31 and ~34 MET Hours per day, respectively) was around 18 months. The maximum effect was reached at an average of 11,247 steps and 34.7 daily MET‐Hours (Figure 1). The effects of physical activity dose on GrimAge acceleration did not differ between men and women (Figure S1; β_Steps × Sex_ = 0.068, 95%CI = [−0.389; 0.525], *p* = 0.769; β_METs × Sex_ = −0.052, 95% CI = [−0.509; 0.404], *p* = 0.823). Additional proportion of time spent in moderate‐to‐vigorous physical activities (MVPA) was also associated with lower GrimAge acceleration (Table 2a). The effect of MVPA on GrimAge acceleration was also non‐linear and was strongest at an average of 5.9% or 1.5 h of daily MVPA (Figure 1). The effect of MVPA on GrimAge acceleration did not differ between men and women (Figure S1; β_MVPA × Sex_ = 0.088, 95%CI = [−0.370; 0.545], *p* = 0.708). To examine whether the non‐linear shape of the association was driven by a few participants with extreme values, we additionally ran a sensitivity analysis excluding participants with high leverage points. After excluding 74 participants with high leverage points, we observed similar effect estimates (Figure S2).

**TABLE 2:** Effect estimates of the association between physical activity and epigenetic ageing.

Term	ß [95%CI]	*p*‐value	*n*	Sample
(*a*) *Main effect estimates for GrimAge acceleration*				
Step Count (linear)	−0.38 [−0.65; −0.12]	0.005	3567	Total Sample
Step Count (quadratic)	0.25 [0.12; 0.39]	<0.001	3567	Total Sample
MET‐Hours (linear)	−0.28 [−0.53; −0.02]	0.032	3567	Total Sample
MET‐Hours (quadratic)	0.24 [0.10; 0.39]	<0.001	3567	Total Sample
% Light Intensity (linear)	−0.17 [−0.42; 0.09]	0.197	3567	Total Sample
% Light Intensity (quadratic)	0.13 [−0.04; 0.29]	0.132	3567	Total Sample
% Moderate‐to‐Vigorous Intensity (linear)	−0.31 [−0.58; −0.04]	0.023	3567	Total Sample
% Moderate‐to‐Vigorous Intensity (quadratic)	0.23 [0.10; 0.37]	<0.001	3567	Total Sample
% Sedentary (linear)	0.16 [−0.09; 0.40]	0.206	3567	Total Sample
% Sedentary (quadratic)	0.12 [−0.05; 0.28]	0.163	3567	Total Sample
(*b*) *Effect estimates of the exploratory analysis for PhenoAge acceleration*				
Step Count (linear)	−0.28 [−0.50; −0.07]	0.010	3567	Total Sample
Step Count (quadratic)	0.17 [0.06; 0.27]	0.003	3567	Total Sample
MET‐Hours (linear)	−0.14 [−0.35; 0.06]	0.178	3567	Total Sample
MET‐Hours (quadratic)	0.13 [0.01; 0.24]	0.033	3567	Total Sample
% Light Intensity (linear)	−0.11 [−0.32; 0.10]	0.293	3567	Total Sample
% Light Intensity (quadratic)	−0.13 [−0.26; 0.01]	0.060	3567	Total Sample
% Moderate‐to‐Vigorous Intensity (linear)	−0.26 [−0.47; −0.04]	0.021	3567	Total Sample
% Moderate‐to‐Vigorous Intensity (quadratic)	0.14 [0.03; 0.25]	0.012	3567	Total Sample
% Sedentary (linear)	0.16 [−0.04; 0.36]	0.110	3567	Total Sample
% Sedentary (quadratic)	−0.09 [−0.22; 0.04]	0.178	3567	Total Sample
(*c*) *Effect estimates of the exploratory analysis for Horvath's age acceleration*				
Step Count (linear)	0.06 [−0.13; 0.25]	0.560	3567	Total Sample
Step Count (quadratic)	0.05 [−0.04; 0.15]	0.267	3567	Total Sample
MET‐Hours (linear)	0.20 [0.02; 0.38]	0.029	3567	Total Sample
MET‐Hours (quadratic)	−0.01 [−0.11; 0.10]	0.893	3567	Total Sample
% Light Intensity (linear)	−0.10 [−0.28; 0.08]	0.295	3567	Total Sample
% Light Intensity (quadratic)	−0.06 [−0.18; 0.05]	0.283	3567	Total Sample
% Moderate‐to‐Vigorous Intensity (linear)	0.11 [−0.08; 0.30]	0.251	3567	Total Sample
% Moderate‐to‐Vigorous Intensity (quadratic)	0.04 [−0.06; 0.13]	0.471	3567	Total Sample
% Sedentary (linear)	0.05 [−0.12; 0.23]	0.565	3567	Total Sample
% Sedentary (quadratic)	−0.08 [−0.20; 0.04]	0.182	3567	Total Sample
(*d*) *Effect estimates of the exploratory analysis for Hannum's age acceleration*				
Step Count (linear)	−0.06 [−0.24; 0.12]	0.509	3567	Total Sample
Step Count (quadratic)	0.06 [−0.03; 0.15]	0.217	3567	Total Sample
MET‐Hours (linear)	0.11 [−0.05; 0.28]	0.183	3567	Total Sample
MET‐Hours (quadratic)	−0.01 [−0.10; 0.09]	0.901	3567	Total Sample
% Light Intensity (linear)	−0.11 [−0.27; 0.06]	0.219	3567	Total Sample
% Light Intensity (quadratic)	0.01 [−0.09; 0.12]	0.806	3567	Total Sample
% Moderate‐to‐Vigorous Intensity (linear)	−0.02 [−0.20; 0.15]	0.805	3567	Total Sample
% Moderate‐to‐Vigorous Intensity (quadratic)	0.04 [−0.05; 0.13]	0.335	3567	Total Sample
% Sedentary (linear)	0.08 [−0.08; 0.24]	0.326	3567	Total Sample
% Sedentary (quadratic)	−0.01 [−0.12; 0.10]	0.881	3567	Total Sample
(*e*) *Effect estimates for GrimAge acceleration of individuals without a cardiovascular event*				
Step Count (linear)	−0.37 [−0.64; −0.10]	0.008	3239	Individuals without a cardiovascular event
Step Count (quadratic)	0.25 [0.12; 0.39]	<0.001	3239	Individuals without a cardiovascular event
MET‐Hours (linear)	−0.25 [−0.50; 0.01]	0.063	3239	Individuals without a cardiovascular event
MET‐Hours (quadratic)	0.24 [0.09; 0.39]	0.002	3239	Individuals without a cardiovascular event
% Light Intensity (linear)	−0.23 [−0.49; 0.03]	0.083	3239	Individuals without a cardiovascular event
% Light Intensity (quadratic)	0.20 [0.03; 0.37]	0.021	3239	Individuals without a cardiovascular event
% Moderate‐to‐Vigorous Intensity (linear)	−0.30 [−0.57; −0.03]	0.032	3239	Individuals without a cardiovascular event
% Moderate‐to‐Vigorous Intensity (quadratic)	0.22 [0.09; 0.36]	0.002	3239	Individuals without a cardiovascular event
% Sedentary (linear)	0.21 [−0.05; 0.46]	0.108	3239	Individuals without a cardiovascular event
% Sedentary (quadratic)	0.20 [0.02; 0.37]	0.025	3239	Individuals without a cardiovascular event

*Note*: Models were adjusted for age, age^2^, sex, education, batch effect, cell proportions, smoking status and season. To allow comparison of effect sizes, we z‐standardised all continuous independent variables.

Abbreviations: MET‐Hours, Metabolic‐Equivalent Hours; 95% CI, 95% confidence interval.

In exploratory analyses examining the association between physical activity and Hannum's age, Horvath's age, and PhenoAge acceleration, we also found greater daily step counts, energy expenditure and time spent in MVPA associated with lower PhenoAge acceleration. The beneficial effects of physical activity dose and MVPA leveled off at higher levels (Table 2b). We did not observe an association between any of the physical activity components and Horvath's and Hannum's age acceleration (Table 2c,d).

### 2.3 Cardiovascular disease risk as mediator

**FIGURE 2:**
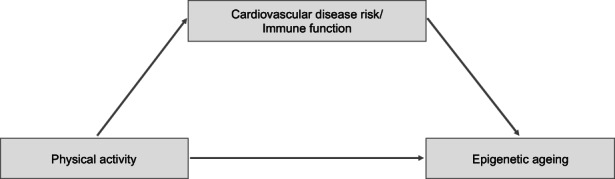
Mediation analysis model.

In our mediation analysis, 3357 eligible participants with available cardiovascular data were included (54.2% women, mean age: 55.4 years, range: 30–94 years) (Table 1). We examined whether cardiovascular disease risk could mediate the association between physical activity (i.e., energy expenditure, step counts and % MVPA) and GrimAge acceleration (Figure 2). We assessed the indirect effect of physical activity on GrimAge acceleration mediated through either the Framingham Risk Score (D'Agostino et al., 2008), the ESC SCORE2 (Hageman et al., 2021), the Assessment of Cardiovascular Disease (ASCVD) Risk Score (Goff et al., 2014) or the sample‐based (factor analysis for mixed data (FAMD)‐derived) cardiovascular disease risk components (Lê et al., 2008). For the sample‐based score, we specifically assessed the mediation effect of the first and second FAMD cardiovascular component, which were predominantly influenced by (1) blood pressure, triglycerides and adiposity measures, and (2) lipoprotein levels, respectively (Figure S3).

We observed that the Framingham Risk Score, the ESC SCORE2, the ASCVD Score and the first FAMD cardiovascular component partially mediated the association between physical activity and GrimAge acceleration (Table 3). The effects of physical activity on GrimAge acceleration depended on physical activity levels. To illustrate the non‐linear association between physical activity and epigenetic age, we report the effects at −1 *SD*, average (0 *SD*) and +1 *SD* levels of physical activity: keeping the covariates at a constant, we found that the direct and indirect effects of physical activity on GrimAge acceleration were strongest at low physical activity quantities (e.g., −1 *SD*) and became progressively weaker at higher levels (i.e., average level and +1 *SD*). Overall, we observed the strongest mediating effects of cardiovascular risk scores for average daily MET‐Hours and step counts. For instance, at one standard deviation below the mean (i.e., 32.6 MET‐Hours and 5570 steps, respectively), 50.1% and 22.2% of the effects of MET‐Hours and step counts on GrimAge acceleration were mediated by the Framingham Risk Score. At the mean (i.e., 34.0 MET‐Hours and 8850 steps, respectively), 40.2% and 27.3% of the effects were mediated by the Framingham Risk Score, with an indirect effect of −0.020 and −0.019 on GrimAge acceleration, respectively. The second FAMD component did not mediate the association between physical activity and GrimAge acceleration (Table 3).

**TABLE 3:** Direct and indirect effects of physical activity on epigenetic ageing mediated by cardiovascular disease risk factors and immune function, while keeping the covariates at a constant.

Mediator	Predictor	Indirect effect			Direct effect			Total effect			Percentage mediated		
		−1 *SD* [95% CI]	Average [95% CI]	+1 *SD* [95% CI]	−1 *SD* [95% CI]	Average [95% CI]	+1 *SD* [95% CI]	−1 *SD* [95% CI]	Average [95% CI]	+1 *SD* [95% CI]	−1 *SD*	Average	+1 *SD*
FAMD cardiovascular disease component 1	MET‐Hours	NA^a^	−0.0082**	NA^a^	−0.1283***	−0.0550**	0.0183	−0.1365***	−0.0632***	0.0101	6.04%	13.04%	NA^b^
			[−0.0146; −0.0023]		[−0.2064; −0.0541]	[−0.0923; −0.0145]	[−0.0262; 0.0616]	[−0.2126; −0.0619]	[−0.1000; −0.0245]	[−0.0356; 0.0547]			
	Step Count	NA^a^	−0.0077*	NA^a^	−0.1505***	−0.0722***	0.0060	−0.1582***	−0.0799***	−0.0016	4.86%	9.61%	NA^b^
			[−0.0140; −0.0020]		[−0.2199; −0.0743]	[−0.1112; −0.0307]	[−0.0345; 0.0441]	[−0.2269; −0.0833]	[−0.1172; −0.0388]	[−0.0437; 0.0366]			
	% MVPA	NA^a^	−0.0071**	NA^a^	−0.1367***	−0.0633**	0.0102	−0.1438***	−0.0704***	0.0031	4.95%	10.11%	NA^b^
			[−0.0129; −0.0021]		[−0.2048; −0.0597]	[−0.1013; −0.0216]	[−0.0315; 0.0490]	[−0.2124; −0.0694]	[−0.1072; −0.0286]	[−0.0389; 0.0418]			
FAMD cardiovascular disease component 2	MET‐Hours	−0.0044	−0.0029	−0.0014	−0.1336***	−0.0607**	0.0122	−0.1380***	−0.0636***	0.0108	3.18%	4.51%	NA^b^
		[−0.0117; 0.0011]	[−0.0075; 0.0008]	[−0.0049; 0.0002]	[−0.2099; −0.0602]	[−0.0975; −0.0222]	[−0.0321; 0.0570]	[−0.2138; −0.0632]	[−0.1003; −0.0249]	[−0.0333; 0.0557]			
	Step Count	−0.0042	−0.0029	−0.0017	−0.1568***	−0.0779***	0.0010	−0.1610***	−0.0808***	−0.0007	2.60%	3.62%	NA^b^
		[−0.0113; 0.0011]	[−0.0077; 0.0008]	[−0.0053; 0.0003]	[−0.2254; −0.0842]	[−0.1153; −0.0368]	[−0.0405; 0.0381]	[−0.2285; −0.0889]	[−0.1185; −0.0398]	[−0.0421; 0.0378]			
	% MVPA	−0.0039	−0.0028	−0.0018	−0.1429***	−0.0685***	0.0059	−0.1468***	−0.0713***	0.0041	2.63%	3.97%	NA^b^
		[−0.0104; 0.0004]	[−0.0075; 0.0005]	[−0.0054; 0.0002]	[−0.2132; −0.0699]	[−0.1060; −0.0278]	[−0.0360; 0.0437]	[−0.2145; −0.0732]	[−0.1083; −0.0302]	[−0.0378; 0.0428]			
Framingham Heart Study cardiovascular score	MET‐Hours	−0.0292***	−0.0195***	−0.0098**	NA^a^	−0.0291	NA^a^	−0.0583***	−0.0486**	−0.0389*	50.10%	40.15%	25.25%
		[−0.0471; −0.0155]	[−0.0292; −0.0114]	[−0.0168; −0.0052]		[−0.0622; 0.0044]		[−0.0910; −0.0215]	[−0.0806; −0.0164]	[−0.0716; −0.0062]			
	Step Count	−0.0268***	−0.0186***	−0.0104***	−0.0942**	−0.0497*	−0.0052	−0.1210***	−0.0683***	−0.0156	22.15%	27.26%	66.88%
		[−0.0431; −0.0141]	[−0.0281; −0.0104]	[−0.0168; −0.0056]	[−0.1667; −0.0237]	[−0.0927; −0.0118]	[−0.0344; 0.0279]	[−0.1924; −0.0513]	[−0.1098; −0.0294]	[−0.0449; 0.0158]			
	% MVPA	−0.0249***	−0.0176***	−0.0104***	−0.0873*	−0.0450*	−0.0028	−0.1122**	−0.0626**	−0.0131	22.19%	28.13%	79.02%
		[−0.0412; −0.0125]	[−0.0270; −0.0098]	[−0.0171; −0.0055]	[−0.1580; −0.0183]	[−0.0868; −0.0065]	[−0.0339; 0.0318]	[−0.1823; −0.0409]	[−0.1041; −0.0225]	[−0.0446; 0.0206]			
ESC SCORE2	MET‐Hours	−0.0283***	−0.0201***	−0.0119***	−0.1090**	−0.0430*	0.0230	−0.1373***	−0.0631***	0.0112	20.60%	31.84%	NA^b^
		[−0.0414; −0.0177]	[−0.0280; −0.0141]	[−0.0175; −0.0071]	[−0.1816; −0.0425]	[−0.0806; −0.0080]	[−0.0214; 0.0723]	[−0.2111; −0.0708]	[−0.1010; −0.0281]	[−0.0331; 0.0594]			
	Step Count	−0.0308***	−0.0212***	−0.0116***	−0.1287***	−0.0586**	0.0115	−0.1594***	−0.0798***	−0.0001	19.31%	26.59%	NA^b^
		[−0.0445; −0.0197]	[−0.0294; −0.0145]	[−0.0177; −0.0071]	[−0.2071; −0.0608]	[−0.0976; −0.0208]	[−0.0274; 0.0553]	[−0.2357; −0.0904]	[−0.1203; −0.0419]	[−0.0395; 0.0423]			
	% MVPA	−0.0287***	−0.0197***	−0.0106***	−0.1174***	−0.0509**	0.0156	−0.1462***	−0.0706***	0.0050	19.67%	27.85%	NA^b^
		[−0.0420; −0.0184]	[−0.0276; −0.0130]	[−0.0156; −0.0056]	[−0.1923; −0.0486]	[−0.0892; −0.0120]	[−0.0229; 0.0598]	[−0.2223; −0.0763]	[−0.1095; −0.0306]	[−0.0336; 0.0501]			
ASCVD SCORE	MET‐Hours	NA^a^	−0.0038*	NA^a^	−0.1301***	−0.0566**	0.0170	−0.1339***	−0.0604**	0.0132	2.84%	6.30%	NA^b^
			[−0.0080; −0.0009]		[−0.2069; −0.0500]	[−0.0941; −0.0142]	[−0.0321; 0.0645]	[−0.2105; −0.0560]	[−0.0975; −0.0176]	[−0.0370; 0.0604]			
	Step Count	−0.0076*	−0.0052*	−0.0027*	−0.1486***	−0.0720***	0.0047	−0.1563***	−0.0771***	0.0020	4.88%	6.72%	NA^b^
		[−0.0176; −0.0017]	[−0.0113; −0.0011]	[−0.0061; −0.0006]	[−0.2186; −0.0717]	[−0.1118; −0.0282]	[−0.0393; 0.0464]	[−0.2260; −0.0802]	[−0.1165; −0.0360]	[−0.0425; 0.0432]			
	% MVPA	−0.0074*	−0.0050*	−0.0026*	−0.1362***	−0.0635**	0.0091	−0.1435***	−0.0685***	0.0065	5.13%	7.26%	NA^b^
		[−0.0175; −0.0017]	[−0.0108; −0.0012]	[−0.0058; −0.0006]	[−0.2104; −0.0613]	[−0.1014; −0.0247]	[−0.0357; 0.0523]	[−0.2161; −0.0667]	[−0.1053; −0.0281]	[−0.0367; 0.0516]			
PCA immune function component 1	MET‐Hours	NA^a^	−0.0045	NA^a^	−0.1306***	−0.0633***	0.0040	−0.1352***	−0.0678***	0.0005	3.36%	6.69%	NA^b^
			[−0.0106; 0.0001]		[−0.2121; −0.0684]	[−0.1012; −0.0289]	[−0.0392; 0.0475]	[−0.2183; −0.0711]	[−0.1065; −0.0341]	[−0.0455; 0.0421]			
	Step Count	NA^a^	−0.0059*	NA^a^	−0.1585***	−0.0820***	−0.0055	−0.1645***	−0.0880***	−0.0114	3.61%	6.75%	51.87%
			[−0.0120; −0.0015]		[−0.2328; −0.0951]	[−0.1223; −0.0467]	[−0.0464; 0.0322]	[−0.2414; −0.1013]	[−0.1304; −0.0545]	[−0.0531; 0.0251]			
	% MVPA	NA^a^	−0.0057*	NA^a^	−0.1447***	−0.0724***	0.0001	−0.1504***	−0.0781***	−0.0058	3.81%	7.35%	NA^b^
			[−0.0119; −0.0014]		[−0.2119; −0.0773]	[−0.1114; −0.0354]	[−0.0411; 0.0407]	[−0.2293; −0.0848]	[−0.1193; −0.0433]	[−0.0491; 0.0311]			
PCA immune function component 2	MET‐Hours	NA^a^	−0.0027*	NA^a^	−0.1287***	−0.0643***	0.0002	−0.1314***	−0.0676***	−0.0025	2.05%	4.02%	NA^b^
			[−0.0060; −0.0009]		[−0.2101; −0.0627]	[−0.1031; −0.0304]	[−0.0443; 0.0426]	[−0.2122; −0.0646]	[−0.1048; −0.0326]	[−0.0479; 0.0401]			
	Step Count	NA^a^	−0.0025*	NA^a^	−0.1612***	−0.0852***	−0.0092	−0.1637***	−0.0877***	−0.0117	1.54%	2.87%	21.44%
			[−0.0058; −0.0008]		[−0.2374; −0.0990]	[−0.1276; −0.0506]	[−0.0499; 0.0271]	[−0.2403; −0.1022]	[−0.1290;−0.0537]	[−0.0530; 0.0237]			
	% MVPA	NA^a^	−0.0024*	NA^a^	−0.1462***	−0.0751***	−0.0041	−0.1486***	−0.0775***	−0.0065	1.61%	3.09%	36.90%
			[−0.0056; −0.0007]		[−0.2232; −0.0801]	[−0.1170; −0.0393]	[−0.0458; 0.0327]	[−0.2252; −0.0825]	[−0.1185; −0.0418]	[−0.0473; 0.0307]			

*Note*: Significance levels: *p ≤ 0.05, **p ≤ 0.01, ***p ≤ 0.001.

Abbreviations: FAMD, factor analysis for mixed data; MET‐Hours, Metabolic‐Equivalent Hours; MVPA, moderate‐to‐vigorous physical activity; PCA, principle component analysis; *SD*, standard deviation; 95% CI, 95% confidence interval.

^a^ Models with only linear effect.

^b^ Models with opposite direct and indirect effects.

### 2.4 Immune function as mediator

In addition, we investigated whether changes in immune function could mediate the association between physical activity and epigenetic ageing (Figure 2). Using principle component analysis, and based on DNA methylation levels, we extracted the sample‐based first and second immune function composite components based on 12 leukocyte subtypes (Salas et al., 2022). Whereas the first component heavily weighted the proportion of neutrophils, as well as (though to a lesser extent) naïve B cells and CD4T+ T cells (naïve CD4T cells and memory CD4T cells), the second component was largely influenced by CD8T+ T cells (memory CD8T cells, naïve CD8T cells, and natural killer cells) (Figure S4). The immune function composite components were only weakly correlated with the cardiovascular disease risk scores and composite components (e.g., r_Immune1 Framingham_ = −0.22; r_Immune2 Framingham_ = 0.11; r_Immune1 ESC Score_ = −0.19; r_Immune2 ESC Score_ = 0.11; r_Immune1 ASCVD_ = −0.15; r_Immune2 ASCVD_ = 0.06).

We observed that the first and second immune function composite components partially mediated the association between physical activity and GrimAge acceleration. However, the mediation effects were generally smaller compared to those of cardiovascular risk factors (Table 3). Whereas the Framingham Risk Score mediated up to ~50% of the effects of physical activity on GrimAge acceleration, both the first and second immune function composite components mediated only up to ~7% of the effects of physical activity on GrimAge acceleration. We found that the direct effect of physical activity on GrimAge acceleration was strongest at low physical activity quantities (e.g., −1 *SD*) and lessened at the higher end of the physical activity spectrum (i.e., average level and +1 *SD*).

### 2.5 Reversed mediation: GrimAge acceleration as mediator

In exploratory analyses, we examined whether GrimAge acceleration could mediate some of the effects of physical activity on cardiovascular disease risk. We observed relatively weak mediating effects of GrimAge acceleration (Table S2). Overall, GrimAge acceleration mediated up to ~10% of the effect of physical activity on cardiovascular disease risk.

### 2.6 Sensitivity analysis in individuals without a cardiovascular event

We repeated the polynomial regression and mediation analysis of cardiovascular disease risk in a subset of individuals without a prior cardiovascular event. In these individuals, the effects of all physical activity components (step counts, energy expenditure, %light intensity activities, %moderate‐to‐vigorous intensity activities and %sedentary activities) were significantly associated with GrimAge acceleration (Table 2e). Higher physical activity dose and intensity were associated with slower epigenetic ageing. The effects of physical activity were again most pronounced at the lower end of the physical activity spectrum, with the exception of the effects of %sedentary activities. A higher proportion of %sedentary activities was associated with faster epigenetic ageing and the effect was stronger at higher %sedentary levels (Figure S5). Compared to other intensities, the effect of %moderate‐to‐vigorous intensity activities was strongest.

Mediation analysis in individuals without a prior cardiovascular event largely replicated the results based on the entire sample (Table 4). The ESC score, Framingham Risk Score and ASCVD Score partially mediated the association between average daily energy expenditure, step counts, %MVPA and GrimAge acceleration. For %light‐intensity activities and %sedentary activities, we found that the ESC score and Framingham Risk Score fully mediated the association with GrimAge acceleration, whereas we did not observe a mediation effect for the ASCVD Score. Moreover, the first FAMD component only fully mediated the association between %light‐intensity activities and GrimAge acceleration. Also here, we did not observe a mediation effect through the second FAMD component.

**TABLE 4:** Direct and indirect effects of physical activity on epigenetic ageing mediated by cardiovascular disease risk in individuals without a prior cardiovascular event, while keeping the covariates at a constant.

Mediator	Predictor	Indirect effect			Direct effect			Total effect			Mediated effect		
		−1 *SD* [95% CI]	Average [95% CI]	+1 *SD* [95% CI]	−1 *SD* [95% CI]	Average [95% CI]	+1 *SD* [95% CI]	−1 *SD* [95% CI]	Average [95% CI]	+1 *SD* [95% CI]	−1 *SD*	Average	+1 *SD*
FAMD cardiovascular disease component 1	MET‐Hours	NA^a^	−0.0062	NA^a^	−0.1296**	−0.0561**	0.0173	−0.1358***	−0.0623**	0.0112	4.54%	9.90%	NA^b^
			[−0.0129; 0.0006]		[−0.2061; −0.0514]	[−0.0957; −0.0167]	[−0.0310; 0.0691]	[−0.2106; −0.0586]	[−0.1030; −0.0249]	[−0.0356; 0.0630]			
	Step Count	NA^a^	−0.0056	NA^a^	−0.1554***	−0.0755***	0.0045	−0.1610***	−0.0810***	−0.0011	3.45%	6.86%	NA^b^
			[−0.0122; 0.0006]		[−0.2363; −0.0839]	[−0.1181; −0.0351]	[−0.0370; 0.0515]	[−0.2399; −0.0920]	[−0.1248; −0.0437]	[−0.0430; 0.0449]			
	% MVPA	NA^a^	−0.0051	NA^a^	−0.1407***	−0.0668**	0.0071	−0.1458***	−0.0719***	0.0019	3.53%	7.15%	NA^b^
			[−0.0113; 0.0002]		[−0.2252; −0.0726]	[−0.1097; −0.0260]	[−0.0339; 0.0559]	[−0.2291; −0.0789]	[−0.1157; −0.0336]	[−0.0396; 0.0506]			
	% Light Intensity	NA^a^	−0.0060*	NA^a^	NA^a^	−0.0165	NA^a^	NA^a^	−0.0226	NA^a^	NA^a^	26.55%	NA^a^
			[−0.0122; −0.0003]			[−0.0525; 0.0253]			[−0.0566; 0.0187]				
	% Sedentary	NA^a^	0.0066	NA^a^	−0.0251	0.0331	0.0913*	−0.0186	0.0397*	0.0979*	NA^b^	16.52%	6.69%
			[−0.0002; 0.0133]		[−0.0770; 0.0309]	[−0.0111; 0.0714]	[0.0154; 0.1690]	[−0.0727; 0.0388]	[−0.0031; 0.0757]	[0.0239; 0.1738]			
FAMD cardiovascular disease component 2	MET‐Hours	NA^a^	−0.0020	NA^a^	−0.1339***	−0.0604**	0.0132	−0.1359***	−0.0623**	0.0112	1.46%	3.18%	NA^b^
			[−0.0052; 0.0003]		[−0.2070; −0.0558]	[−0.1015; −0.0239]	[−0.0339; 0.0655]	[−0.2101; −0.0582]	[−0.1030; −0.0247]	[−0.0353; 0.0637]			
	Step Count	NA^a^	−0.0020	NA^a^	−0.1602***	−0.0794***	0.0013	−0.1622***	−0.0814***	−0.0006	1.23%	2.44%	NA^b^
			[−0.0054; 0.0003]		[−0.2371; −0.0920]	[−0.1215; −0.0418]	[−0.0407; 0.0464]	[−0.2409; −0.0935]	[−0.1242; −0.0441]	[−0.0426; 0.0454]			
	% MVPA	NA^a^	−0.0020	NA^a^	−0.1456***	−0.0706	0.0045	−0.1476***	−0.0725***	0.0025	1.33%	2.71%	NA^b^
			[−0.0054; 0.0001]		[−0.2245; −0.0763]	[−0.1130; −0.0327]	[−0.0368; 0.0525]	[−0.2272; −0.0789]	[−0.1160; −0.0340]	[−0.0392; 0.0516]			
	% Light Intensity	−0.0048	−0.0022	0.0005	NA^a^	−0.0210	NA^a^	−0.0258	−0.0232	−0.0205	18.56%	9.27%	NA^b^
		[−0.0128; 0.0002]	[−0.0056; 0.0001]	[−0.0008; 0.0039]		[−0.0550; 0.0195]		[−0.0610; 0.0150]	[−0.0578; 0.0172]	[−0.0552; 0.0198]			
	% Sedentary	−0.0006	0.0020	0.0049	−0.0188	0.0377	0.0942*	−0.0194	0.0398*	0.0991*	2.97%	5.44%	4.95%
		[−0.0039; 0.0007]	[−0.0004; 0.0055]	[−0.0014; 0.0130]	[−0.0725; 0.0388]	[−0.0045; 0.0742]	[0.0178; 0.1719]	[−0.0730; 0.0379]	[−0.0032; 0.0755]	[0.0248; 0.1749]			
Framingham Heart Study cardiovascular score	MET‐Hours	−0.0297***	−0.0200***	−0.0102**	NA^a^	−0.0250	NA^a^	−0.0548**	−0.0450**	−0.0353*	54.28%	44.38%	29.01%
		[−0.0501; −0.0159]	[−0.0317; −0.0114]	[−0.0182; −0.0046]		[−0.0600; 0.0071]		[−0.0896; −0.0189]	[−0.0795; −0.0122]	[−0.0700; −0.0027]			
	Step Count	−0.0234**	−0.0172***	−0.0109***	−0.0873*	−0.0464*	−0.0056	−0.1108**	−0.0636**	−0.0165	21.17%	27.00%	66.22%
		[−0.0427; −0.0117]	[−0.0286; −0.0096]	[−0.0181; −0.0051]	[−0.1535; −0.0104]	[−0.0865; −0.0054]	[−0.0378; 0.0273]	[−0.1753; −0.0317]	[−0.1001; −0.0197]	[−0.0492; 0.0147]			
	% MVPA	−0.0217**	−0.0160***	−0.0103**	−0.0796*	−0.0423	−0.0050	−0.1013**	−0.0583**	−0.0152	21.42%	27.44%	67.76%
		[−0.0396; −0.0102]	[−0.0267; −0.0084]	[−0.0174; −0.0047]	[−0.1439; −0.0037]	[−0.0813; 0.0028]	[−0.0360; 0.0290]	[−0.1616; −0.0264]	[−0.0936; −0.0106]	[−0.0454; 0.0186]			
	% Light Intensity	−0.0249**	−0.0146***	−0.0043	NA^a^	−0.0140	NA^a^	−0.0389*	−0.0286	−0.0183	64.06%	51.10%	23.51%
		[−0.0461; −0.0120]	[−0.0249; −0.0078]	[−0.0122; 0.0024]		[−0.0492; 0.0208]		[−0.0729; −0.0009]	[−0.0627; 0.0062]	[−0.0535; 0.0166]			
	% Sedentary	0.0046	0.0165***	0.0284**	NA^a^	0.0194	NA^a^	0.0240	0.0359*	0.0478*	19.00%	45.94%	59.43%
		[−0.0024; 0.0125]	[0.0092; 0.0269]	[0.0153; 0.0508]		[−0.0160; 0.0558]		[−0.0112; 0.0620]	[0.0016; 0.0720]	[0.0121; 0.0835]			
ESC SCORE2	MET‐Hours	−0.0235***	−0.0182***	−0.0130***	−0.1105**	−0.0429*	0.0247	−0.1340***	−0.0611**	0.0117	17.55%	29.84%	NA^b^
		[−0.0380; −0.0132]	[−0.0270; −0.0118]	[−0.0190; −0.0078]	[−0.1836; −0.0299]	[−0.0841; −0.0018]	[−0.0258; 0.0768]	[−0.2087; −0.0529]	[−0.1019; −0.0192]	[−0.0393; 0.0605]			
	Step Count	−0.0235***	−0.0179***	−0.0123***	−0.1359***	−0.0617**	0.0124	−0.1594***	−0.0797***	0.0004	14.77%	22.51%	NA^b^
		[−0.0384; −0.0132]	[−0.0270; −0.0112]	[−0.0180; −0.0074]	[−0.2050; −0.0568]	[−0.0994; −0.0173]	[−0.0338; 0.0553]	[−0.2288; −0.0805]	[−0.1195; −0.0356]	[−0.0450; 0.0418]			
	% MVPA	−0.0215***	−0.0162***	−0.0109***	−0.1237**	−0.0547*	0.0144	−0.1452***	−0.0709***	0.0035	14.83%	22.87%	NA^b^
		[−0.0359; −0.0118]	[−0.0248; −0.0098]	[−0.0165; −0.0061]	[−0.1931; −0.0458]	[−0.0934; −0.0123]	[−0.0322; 0.0579]	[−0.2133; −0.0657]	[−0.1098; −0.0276]	[−0.0421; 0.0478]			
	% Light Intensity	−0.0252***	−0.0173***	−0.0094**	NA^a^	−0.0092	NA^a^	−0.0344	−0.0265	−0.0186	73.17%	65.20%	50.48%
		[−0.0398; −0.0146]	[−0.0261; −0.0112]	[−0.0164; −0.0039]		[−0.0424; 0.0263]		[−0.0715; 0.0020]	[−0.0606; 0.0091]	[−0.0527; 0.0172]			
	% Sedentary	0.0095**	0.0192***	0.0288***	NA^a^	0.0140	NA^a^	−0.0235	0.0332	0.0428*	40.45%	57.77%	67.28%
		[0.0040; 0.0165]	[0.0126; 0.0279]	[0.0167; 0.0455]		[−0.0234; 0.0480]		[−0.0133; 0.0578]	[−0.0024; 0.0673]	[0.0074; 0.0798]			
ASCVD Score	MET‐Hours	NA^a^	−0.0035*	NA^a^	−0.1296***	−0.0561**	0.0175	−0.1331***	−0.0596**	0.0140	2.64%	5.89%	NA^b^
			[−0.0082; −0.0010]		[−0.2036; −0.0510]	[−0.0968; −0.0116]	[−0.0272; 0.0686]	[−0.2072; −0.0550]	[−0.0998; −0.0155]	[−0.0307; 0.0650]			
	Step Count	−0.0066	−0.0047*	−0.0028	−0.1518***	−0.0735***	0.0048	−0.1585***	−0.0783***	0.0019	4.19%	6.04%	NA^b^
		[−0.0157; −0.0016]	[−0.0105; −0.0012]	[−0.0068; −0.0008]	[−0.2206; −0.0765]	[−0.1124; −0.0292]	[−0.0373; 0.0497]	[−0.2279; −0.0848]	[−0.1165; −0.0339]	[−0.0402; 0.0472]			
	% MVPA	NA^a^	−0.0035*	NA	−0.1386***	−0.0656**	0.0073	−0.1421***	−0.0691***	0.0038	2.47%	5.07%	NA^b^
			[−0.0080; −0.0009]		[−0.2135; −0.0673]	[−0.1039; −0.0214]	[−0.0344; 0.0561]	[−0.2180; −0.0725]	[−0.1082; −0.0249]	[−0.0393; 0.0508]			
	% Light Intensity	NA^a^	−0.0015	NA^a^	NA^a^	−0.0204	NA^a^	NA^a^	−0.0219	NA^a^	NA^a^	6.85%	NA^a^
			[−0.0047; 0.0003]			[−0.0545; 0.0130]			[−0.0561; 0.0120]				
	% Sedentary	NA^a^	0.0024	NA^a^	−0.0216	0.0360	0.0936*	−0.0193	0.0384*	0.0960*	NA^b^	6.14%	2.50%
			[0.0004; 0.0063]		[−0.0747; 0.0309]	[−0.0018; 0.0737]	[0.0183; 0.1701]	[−0.0732; 0.0331]	[0.0007; 0.0763]	[0.0213; 0.1746]			

*Note*: Significance levels: *p ≤ 0.05, **p ≤ 0.01, ***p ≤ 0.001.

Abbreviations: FAMD, factor analysis for mixed data; MET‐Hours, Metabolic‐Equivalent Hours; MVPA, %Moderate‐to‐Vigorous Physical Activity; *SD*, standard deviation; 95% CI, 95% confidence interval.

^a^ Models with only linear effect.

^b^ Models with opposite direct and indirect effects.

### 2.7 Mediation analysis using a negative control variable

To test the robustness of the mediation results, we also ran mediation analyses with olfactory performance as a negative control variable. We found that olfactory performance did not mediate the association between physical activity and GrimAge acceleration (Table S3).

### 2.8 EWAS of physical activity and functional analyses

**FIGURE 3:**
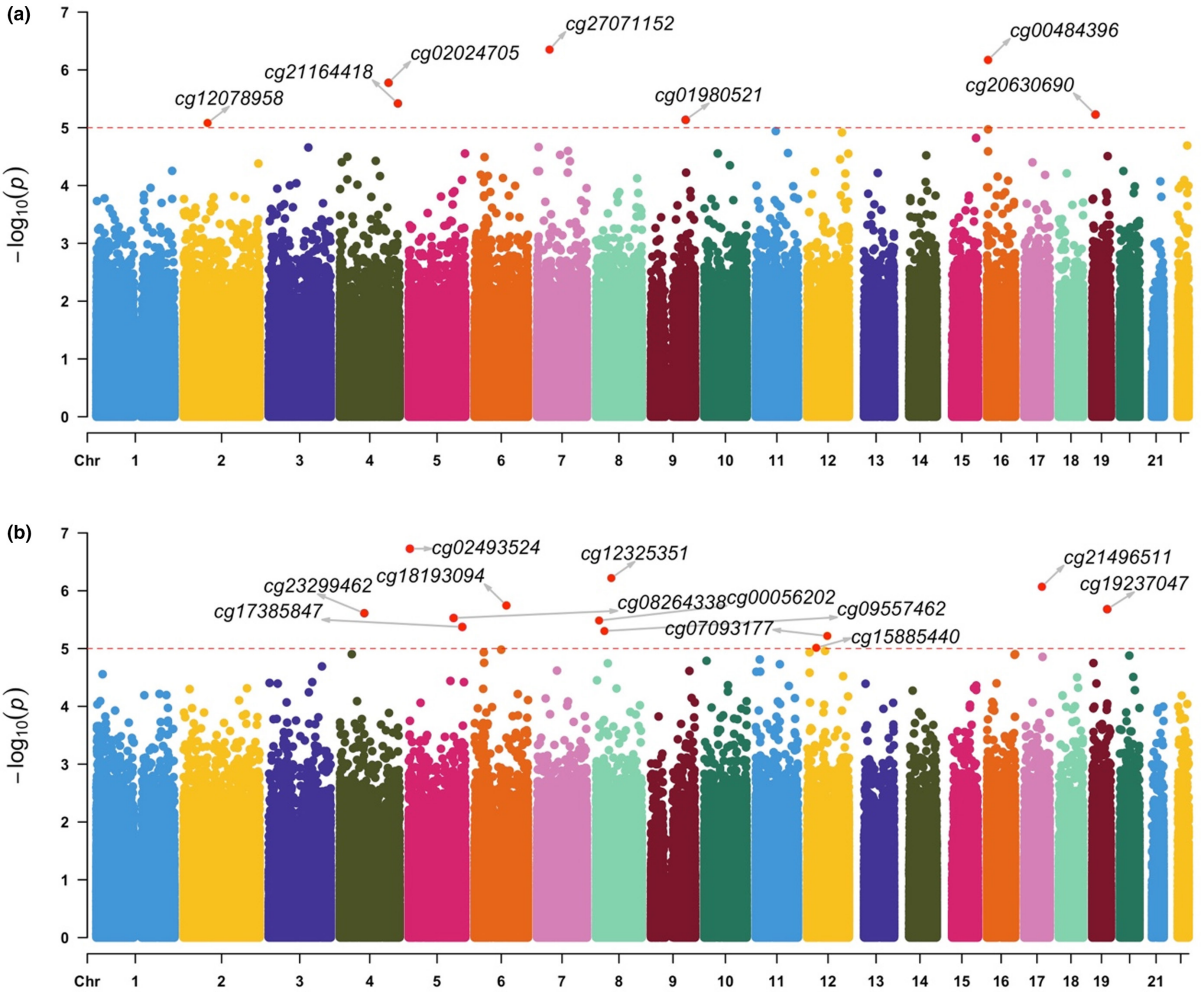
EWAS results of physical activity components. Manhatten plots of the epigenome‐wide association study (EWAS) results for (a) % average daily time spent in moderate‐to‐vigorous activities and (b) average daily energy expenditure in MET‐Hours. The *x*‐axis depicts sites ordered by chromosomal position with the respective ‐log_10_ *p*‐value on the *y*‐axis. The horizontal lines represent the level of significance, with the red horizontal dashed line at the nominal significant level (*p*‐value *p* < 1E‐05).

At the nominally significant threshold (defined as *p* < 1 × 10^−5^), we identified 7 CpGs associated with %time spent in MVPA (Figure 3a and Table S4a), and 12 CpGs associated with MET‐Hours (Figure 3b and Table S4b). However, we did not discover any epigenome‐wide significant CpGs (all FDR > 0.05). We did not identify any genomic inflation of the test statistics (Figure S7).

We performed lookup analyses of all the CpG sites and the mapped genes for each of the CpGs, showing an association with %MVPA and MET‐Hours at the nominally significant level using the EWAS Catalog (http://ewascatalog.org/), EWAS Atlas (https://ngdc.cncb.ac.cn/ewas/atlas) and GWAS catalog (https://www.ebi.ac.uk/gwas/) (Table S4a). %MVPA and MET‐Hours associated CpGs have been previously linked to immune function (i.e., CD24 on IgD+ CD24+ B cell, CD24 on memory B cell, blood cell counts), cardiometabolic traits (i.e., waist circumference, body mass index (BMI), blood pressure, QT interval, ischemic stroke, myocardial infarction) and other ageing‐related traits (i.e., cognitive function, neuritic plaques, white matter hyperintensities, type 2 diabetes). Of note, cg18193094, positionally mapped to the glutamate ionotropic receptor kainite type subunit 2 (*GRIK2*) gene, which has previously been linked to inter‐individual differences in heart rate increase and recovery during and after exercise (Verweij et al., 2018).

Over‐representation analysis using Webgestalt GWAS catalog (https://www.ebi.ac.uk/gwas/) based on the nearest genes of the CpG sites showing an association with energy expenditure and %MVPA at a nominally significant level did not yield significant Gene Ontology (GO) terms after adjustment of multiple comparisons (data not shown). However, gene set enrichment analysis using clusterProfiler (Wu et al., 2021) based on a significance‐ranked list of all genes (i.e., from the lowest to the highest *p*‐value of the corresponding CpG site), identified a large number of GO terms related to biological processes, molecular function and cellular components (FDR < 0.05). We observed that the identified genes were particularly enriched in pathways related to regulation of vascular endothelial function, cell proliferation, interaction and signalling and sensory development and perception (Tables S5 and S6).

To assess potential genetic confounding, we used mQTLdb to examine whether there were previously identified methylation quantitative trait loci (mQTLs) for the CpGs that were found to be associated with MVPA and MET‐Hours (Gaunt et al., 2016). There were no mQTLs for 5 CpGs that were associated with MVPA, but several mQTLs were previously identified for cg27071152 and cg00484396. We also found several mQTLs for cg19237047, cg17385847 and cg09557462 that were associated with MET‐Hours, but not for the other 9 CpGs (Table S4). These mQTLs might have an impact on the methylation levels of these CpGs.

We performed additional sensitivity analyses to assess whether BMI modifies the association between physical activity and methylation levels of the nominally significant CpGs. We found that although the mediation estimates became smaller after BMI adjustment, they remained directionally consistent (Table S7). We did not find any interaction effects between cell types and methylation levels of the nominally significant CpGs on physical activity levels (all *p* values for the interaction terms >0.05).

## 3 DISCUSSION

We found that higher levels of accelerometer‐assessed physical activity were associated with slower epigenetic ageing in the general population, with similar effects in both men and women. Health benefits from engaging in physical activity are well‐documented (World Health Organization, 2020). Lifestyle interventions incorporating physical activity may thus be cost‐effective and easily implementable measures to slow down age‐associated functional decline as reflected in decelerated epigenetic ageing. Importantly, both the absolute and relative effects of physical activity dose and intensity on epigenetic ageing were most pronounced at the lower end of the activity spectrum. Our findings therefore indicate that health benefits of additional physical activity are likely to be greatest in adults leading a sedentary lifestyle.

While previous studies predominantly relied on self‐reported physical activity levels, to the best of our knowledge, our study is the first to assess the effects of detailed accelerometer‐derived physical activity components on epigenetic ageing in adults over a wide age range. In line with previous studies (Kresovich et al., 2021; Lu et al., 2019), we observed that higher physical activity levels are associated with slower GrimAge acceleration. Specifically, we found slower epigenetic ageing to be related to higher energy expenditure, step counts and more time spent in moderate‐to‐vigorous intensity activities. In individuals without a history of cardiovascular disease, also more time spent in light intensity activities was associated with slower GrimAge acceleration, whereas longer sedentary time was associated with faster epigenetic ageing. Nonetheless, when considering different levels of physical activity intensity, also in this subset of individuals moderate‐to‐vigorous intensity physical activity had the strongest effect on epigenetic ageing. Our findings imply that all levels of physical activity may be effective for the prevention of cardiovascular disease, although moderate‐to‐vigorous intensity activities may yield the strongest benefits.

In further exploratory analyses, we examined whether various physical activity components are also associated with other epigenetic clocks such as PhenoAge, Hannum's and Horvath's age. We only observed an association between physical activity and second‐generation (GrimAge and PhenoAge acceleration), but not first‐generation clocks (Hannum's and Horvath's age acceleration), which has also been reported in a recent systematic review (Oblak et al., 2021). Slower PhenoAge acceleration was associated with higher energy expenditure, step counts and more time spent in moderate‐to‐vigorous activities. We found the strongest association between physical activity and GrimAge acceleration, which has been hypothesized to be the prime indicator of age‐associated functional decline across all epigenetic clocks (Lu et al., 2019; McCrory et al., 2021).

The molecular pathways through which physical activity affects epigenetic ageing are thus far poorly understood. To explore the effects of physical activity on methylation status directly, we conducted an epigenome‐wide association study across 850,000 CpG sites. We found methylation status of several CpG sites to show a nominal significant association with time spent in moderate‐to‐vigorous physical activity (*n* = 7 CpGs) and MET‐Hours (*n* = 12 CpGs). Interestingly, the nearest gene to one of these CpGs, *GRIK2*, was also associated with heart rate response and recovery after exercise in a previous study (Verweij et al., 2018), while mutations in *GRIK2* are known to cause several autosomal recessive forms of intellectual disability (https://omim.org/entry/138244). Gene enrichment analysis of the nearest genes across the CpG sites demonstrated that genes, whose methylation levels were most strongly associated with physical activity, were enriched in pathways related to regulation of vascular endothelial function as well as pathways related to nervous system function and health (including synaptic signalling, sensory development and perception, as well as cell proliferation and interaction). These results thus suggest that physical activity may particularly affect neuronal signalling, thereby pointing to a novel molecular basis for previous findings suggesting that physical activity is beneficial for brain function (Silverman & Deuster, 2014; World Health Organization, 2020), an effect that is particularly evident in brain regions with a high oxidative demand (Fox et al., 2022). Larger EWAS of physical activity are required for the identification of additional molecular mechanisms through which physical activity exerts its beneficial health effects.

Several potential pathways have been suggested through which physical activity could exert its positive effects on health. Exercise has been found to minimize inflammation symptoms and oxidative stress and to promote neuroplasticity and growth factor expression (Silverman & Deuster, 2014). It is noteworthy that the negative effects of exercise on inflammatory biomarkers such as adipokines (e.g., interleukin‐6, tumor necrosis factor alpha) and the stimulating effects of exercise on cortisol and adrenaline secretion are most pronounced for prolonged and high intensity exercise bouts (Gleeson et al., 2011; Peake et al., 2005). It has been suggested that the anti‐inflammatory effects of exercise could alter the epigenome and a few small scale studies have indeed linked exercise to the methylation status of genes linked to inflammation, tumor growth, and neuroplasticity (Ferioli et al., 2019). In this study we found that sample‐based immune function composites, which were mainly influenced by naïve B cells, CD4T+ T cells and CD8T+ T cells, partially mediated the effect of physical activity on epigenetic ageing. However, it should be noted that the mediated proportion was small. The reduction of naïve cells and accumulation of activated immune T cells is a well‐established feature of ageing and immunosenescence. Previous studies suggest that the activation of T and NK cells could be major contributors to epigenetic ageing (Jonkman et al., 2022), whereas physical activity could lead to an increased mobilization of T and NK cells and a decreased accumulation of senescent T cells (Duggal et al., 2019). Our findings thus indicate that particularly regular exercise with a moderate‐to‐high intensity level may lead to long lasting changes of the epigenome and reduce epigenetic ageing. The beneficial effects of physical activity on epigenetic ageing could be, in part, ascribed to its positive effects on immunosenenscence. However, further experimental studies are needed to study the immunological mechanisms through which physical activity could act upon epigenetic ageing.

Here, we also present the first study assessing whether well‐established cardiovascular risk factors could mediate the relation between physical activity and epigenetic ageing. Accelerated epigenetic ageing has been associated with a higher cardiovascular disease risk (Joyce et al., 2021). Several studies have found that the effects of physical activity on cardiovascular disease risk is closely linked to DNA methylation changes (Ferrari et al., 2019; Sellami et al., 2021). Indeed, we found that the Framingham Risk Score, which is based on the most important cardiovascular risk factors, the Assessment of Cardiovascular Disease (ASCVD) Risk Score, the most recently published guideline to assess cardiovascular risk by the American College of Cardiology, and the European Society of Cardiology Score (ESC SCORE2), which is scaled to reflect country‐specific cardiovascular risk, partially mediated the association between physical activity and epigenetic ageing. To dissect the relative mediation effects of different cardiovascular risk factors, we also created data‐driven sample‐specific cardiovascular summary measures. Our first sample‐based cardiovascular risk component, which primarily captured the combined effects of blood pressure, adiposity markers, and triglyceride levels, partially mediated the relation between physical activity and epigenetic ageing. In contrast, the second sample‐based cardiovascular component, which primarily captured the effects of lipoprotein levels, did not show a mediation effect. Therefore, our findings indicate that the effects of physical activity on health are preferentially mediated through specific cardiovascular risk factors, especially blood pressure, adiposity, and triglyceride levels.

Our findings substantially extend those of previous studies reporting beneficial effects of physical activity in the prevention and treatment of cardiovascular diseases, and suggest that targeting hypertension, hypertriglyceridemia, and adiposity may be particularly effective in counteracting cardiovascular ageing (Eriksson et al., 1997). Blood pressure, triglycerides, and adiposity markers have all been found to causally affect methylation status (Mendelson et al., 2017; Richard et al., 2017; Wahl et al., 2017), and several adiposity‐related traits have been associated with faster GrimAge acceleration (McCartney et al., 2021). Compared to the Framingham Heart Study sample (D'Agostino et al., 2008), our participants were on average slightly older, were more often treated for hypertension and had lower total cholesterol, but higher HDL levels. Our sample also included more non‐smokers. Given that the Framingham Risk Score weighs hypertension markers and age more than cholesterol levels, this might offer an explanation why we observed a mediation effect of the Framingham Risk Score and the first, but not the second, FAMD cardiovascular component. Nonetheless, further studies are warranted to elucidate the molecular underpinnings of the effects of physical activity on hypertension and adiposity markers, as well as their influence on epigenetic ageing.

### 3.1 Limitations

Several limitations of this study should be noted. First, given the cross‐sectional nature of this study, we could not assess the causality of the association between physical activity and epigenetic ageing. Second, actimetry recordings were conducted outside of the laboratory and in the participants' normal environment. Even though participants' daily activities were recorded during regular activity weeks, participants may have consciously or unconsciously changed their activity pattern during this recording period. Third, while the activPAL accelerometer is highly accurate in identifying changes in postures and intensity categories, distinguishing been different postures, such as car driving versus taking steps, might sometimes be unreliable since the device is attached to the thigh; however, through rigorous quality control of the data, we were able to exclude unreliable measurements. Furthermore, classification of moderate versus high intensity activities with the activPAL proprietary software can be imprecise as the algorithm linearly classifies energy expenditure based on cadence. To reach the classification threshold of vigorous intensity (typically defined as ≥ 6 METs), a minimum cadence of 240 steps/min would be required. However, even athletes rarely reach a cadence of more than 200 steps/min for prolonged periods of time. Fourth, it cannot be ruled out that our findings were partly influenced by selection bias as participants of our study had relatively high education and physical activity levels; however, if anything, this is likely to have resulted in an underestimation of the effects of physical activity on epigenetic ageing.

## 4 CONCLUSION

In conclusion, we demonstrated that higher accelerometer‐assed physical activity levels are associated with slower epigenetic ageing in adults across a wide age range. We observed that the effect of physical activity on epigenetic ageing can largely be attributed to its beneficial effects on cardiovascular health and immune function. Given the expected rapid rise in the prevalence of cardiovascular diseases, exercise regimens focusing on moderate‐to‐high intensity activities could serve as inexpensive, easily actionable and effective preventive lifestyle interventions. Particularly adults leading a sedentary lifestyle may profit from engaging in additional exercise.

## 5 EXPERIMENTAL PROCEDURES

### 5.1 Study population

Our analysis was based on cross‐sectional baseline data from the first 5000 participants of the Rhineland Study (age range = 30–94 years), an ongoing population‐based prospective cohort study (Fox et al., 2022). Invitations to participate in the Rhineland Study are send to inhabitants of two distinct municipal districts in Bonn, Germany, who are 30 years or older. To participate, invitees are required to have a sufficient command of the German language to provide informed consent. Participants complete multiple assessments including questionnaires, blood collection, anthropometric and cardiovascular measurements, and accelerometer attachment. The study was approved by the ethics committee of the University of Bonn, Medical Faculty, and is carried out according to the principles of the Declaration of Helsinki.

In this study, we analysed data of 3567 eligible individuals out of the first 5000 participants of the Rhineland Study (Figure S6). Actimetry recordings were not available for 1005 participants due to the following reasons: refusal to participate (*n* = 71), technical/acquisition failure (*n* = 187) or ineligibility (*n* = 747). Participants were deemed ineligible if they were unable to stand or walk, had an unrepresentative physical activity week and/or were allergic to medical adhesives. Representativeness of the physical activity week was established based on self‐reports. Participants were asked to judge whether they anticipated having a regular, representative activity and rest pattern during the recording time. Reasons for an unrepresentative physical activity week included vacation, untypical work trips, surgery or hospital stays, which could result in shifted and unrepresentative activity and rest patterns. In addition, we also excluded 122 participants with less than 5 valid recording days to achieve reliable physical activity estimates (Aguilar‐Farias et al., 2019). We classified and excluded recording days as invalid based on Winkler and colleagues' proposed criteria: <500 steps/day, ≥95% time spent in one posture, and <10 h estimated waking wear time (Winkler et al., 2016). We visually checked heatmaps of included and excluded recordings and wear diaries to avoid incorrect exclusion. Using a modified z‐score of 3.5, we identified potential outliers and after visual inspection excluded 5 participants with erroneous actimetry recordings. Furthermore, we excluded 60 participants with missing covariate data and 239 participants with missing epigenetic clock data. For the mediation analysis, we additionally excluded 210 participants with missing cardiovascular data.

### 5.2 Physical activity

We used the activPAL3 micro (PAL Technologies, Glasgow, UK) to measure physical activity intensity, step counts and energy expenditure continuously across seven consecutive days. We processed raw data using the proprietary activPAL software suite. Based on a customized version of the “activpalProcessing” package in R version 3.6.3 (The R Foundation), we extracted information on physical activity dose (energy expenditure, step counts) and intensity (sedentary, light intensity, moderate‐to‐vigorous intensity) (Lyden et al., 2017). We calculated weighted daily averages for all physical activity variables, which were adjusted for accelerometer wear time per day. Physical activity dose variables included average weighted daily step counts and average weighted daily energy expenditure as reflected in metabolic equivalents (METs) per hour. We defined physical activity intensity based on posture and energy expenditure across time: average daily % time spent in sedentary (sitting/lying posture), light‐intensity (standing or step‐taking posture and METs <3.0), or moderate‐to‐vigorous (standing or step‐taking posture and METS ≥ 3.0) activities. Further details on the physical activity assessment have been described previously (Fox et al., 2022).

### 5.3 DNA methylation quantification

Genomic DNA was extracted from buffy coat fractions of anti‐coagulated blood samples using Chemagic DNA buffy coat kit (PerkinElmer, Germany) and was subsequently bisulfite converted using the DNA methylation kit according to the manufacturer's instructions. DNA methylation levels were measured using Illumina's Human MethylationEPIC BeadChip. The methylation level for each probe was derived as a beta value representing the fractional level of DNA methylation at that probe. Sample‐level and probe‐level quality control was performed using the ‘minfi’ package in R (version 3.5.0) (Fortin et al., 2017). Samples with sex mismatch or a missing rate at >1% across all probes were excluded. Probes with a missing rate >1% (at a detection *p*‐value >0.01) were also excluded following previously published recommendation guidelines for analyzing methylation data (Wu & Kuan, 2018).

### 5.4 Estimation of epigenetic age acceleration

DNAm Hannum age, Horvath age, PhenoAge and GrimAge was calculated as described previously (Hannum et al., 2013; Horvath, 2013; Levine et al., 2018; Lu et al., 2019). The age acceleration estimators were defined as the residuals (in years) that result from regressing the DNAm age estimates on chronological age.

### 5.5 DNA methylation‐based immune phenotyping

Based on DNA methylation levels, the relative proportion of twelve leukocyte subtypes (basophils, eosinophils, neutrophils, monocytes, naïve B cells, memory B cells, naïve CD4T cells, memory CD4T cells, regulatory T cells, naïve CD8T cells, memory CD8T cells and natural killer cells) was derived using the “FlowSorted.BloodExtended.EPIC” package in R (version 3.6.3, The R Foundation), which is based on a reference‐based deconvolution method described by Salas et al. (2022). We created sample‐based immune profile composites based on principle component analysis of all leukocyte variables, using the first two principle components (Figure S3).

### 5.6 Covariates

The International Standard Classification of Education 2011 (ISCED) was used to categorize participants' highest education levels as low (lower secondary education or below), middle (upper secondary education to undergraduate university level) and high (postgraduate university study). We determined participants' age, sex, smoking status (smokers vs. non‐smokers) and diabetes status (diabetic vs. non‐diabetic) based on self‐report. Missing smoking values were imputed based on cotinine metabolite levels. Individuals with a cotinine level exceeding the non‐smoker sample‐defined 97.5 percentile were classified as smokers. We derived the season of the actimetry recording based on the dates of the recordings. Olfactory performance was assessed with the 12‐item Sniffin' Stick odour identification test and defined as the total number of correctly identified pens (Lu et al., 2021). Anthropometric examinations were performed using a SECA 285 measuring station and SECA 201 measuring tape. Waist‐to‐hip ratio (WHR) was calculated as a ratio of waist circumference to hip circumference and BMI was calculated as weight [kg]/(height [m])^2^. Serum levels of cholesterol, high‐density lipoprotein (HDL), low‐density lipoprotein (LDL) and triglycerides were measured using routine methods at the Clinical Chemistry Laboratory of University Hospital Bonn. Resting blood pressure was measured three times with 10‐min intervals and average systolic blood pressure (SBP) and diastolic blood pressure (DBP) were calculated using the last two measured values. Hypertension was based on regular use of antihypertensive medication, average SBP (≥140 mm Hg) and average DBP (≥90 mm Hg). A cardiovascular event was defined based on self‐reported medical history of myocardial infarction, coronary artery disease, transient ischemic attack (TIA), cardiac insufficiency, peripheral arterial disease, pacemaker placement, stroke, aortic surgery, carotid artery surgery and peripheral artery surgery.

### 5.7 Cardiovascular disease risk score

Based on previously published algorithms, 10‐year cardiovascular disease risk was calculated based on the Framingham Risk Score, the European Society of Cardiology Score (ESC SCORE2) and the Assessment of Cardiovascular Disease (ASCVD) Risk Score (D'Agostino et al., 2008; Goff et al., 2014; Hageman et al., 2021). In addition, we created a sample‐based cardiovascular risk component score based on age, waist‐to‐hip ratio, cholesterol, HDL, LDL, triglycerides and insulin levels, smoking, hypertension and diabetes status as well as SBP and DBP, using factor analysis for mixed data (FAMD) as implemented in the ‘FactoMineR’ package in R (version 3.6.3, The R Foundation) (Lê et al., 2008). We extracted the first two components as summary measures for our sample‐based cardiovascular risk score (Figure S3).

### 5.8 Statistical analysis

Statistical analyses were performed in R (version 3.6.3, The R Foundation). In the sample demographics, we present mean and standard deviation (*SD*) for continuous variables and number and percentage for categorical variables. We assessed differences between included and excluded participants using binomial logistic regression adjusted for age and sex.

To examine the association between physical activity (independent variable) and epigenetic age acceleration (outcome), we used multivariable (polynomial) regression models. We tested for potential non‐linear effects of physical activity on epigenetic ageing by including a quadratic term for physical activity. In addition, we also assessed whether the effects of physical activity on epigenetic age acceleration differed between men and women by including interaction terms between physical activity and sex. To account for residual confounding, we adjusted the models for age, age^2^ as well as for batch effect, cell proportions (CD4T+ T cells, CD8T+ T cells, neutrophils, monocytes and granulocytes), sex, education and smoking status. To allow comparison of effect sizes, we z‐standardised all continuous independent variables. Effect estimates are presented with the corresponding two‐sided 95% confidence intervals. The threshold for statistical significance was set at *p* ≤ 0.05. Visual inspection of the distribution of residuals was performed to evaluate whether model assumptions were met.

In addition, we performed two sensitivity analyses: (1) We re‐ran the polynomial regression models excluding data points with a high leverage (i.e., those observations with values of the independent variables far from those of other independent variables, defined as a hat value exceeding 3 times the average), and (2) We also assessed whether the association between physical activity and epigenetic ageing changed when excluding participants with a prior cardiovascular event.

In follow‐up analysis, we wanted to examine whether the association between physical activity (independent variable) and GrimAge acceleration (outcome) was mediated through immune function and cardiovascular disease risk (mediators). We performed structural equation modelling using the ‘lavaan’ package in R (version 3.6.3, The R Foundation) (Rosseel, 2012). GrimAge acceleration estimates were adjusted for batch effects and cell proportions, while the mediation models were additionally adjusted for age, age^2^, sex, season, and education. Given the non‐linear effect of physical activity on cardiovascular disease risk and epigenetic ageing, we present estimates at the average, 1 *SD* below and 1 *SD* above mean physical activity dose and intensity. Quadratic physical activity terms were only added to the final models if they were statistically significant (*p* ≤ 0.05). In a sensitivity analysis, we re‐examined the mediation effect through cardiovascular disease risk factors while excluding individuals with a prior cardiovascular event. In addition, to test the specificity of the mediation effects, we performed mediation analyses examining whether the effects of physical activity on epigenetic ageing could also be mediated through olfactory performance (negative control variable).

### 5.9 Epigenome‐wide association study (EWAS) of physical activity and functional analyses

We examined the association between physical activity components (independent variables) and DNA methylation level (outcome) using multiple linear regression, while adjusting for age, sex, batch effects, blood cell proportion, the first ten genetic principal components (to account for population stratification), and smoking. As our initial analyses suggested relatively high genomic inflation for the MVPA EWAS, we restricted the EWAS analyses to participants from Caucasian descent (*n* = 3159) which resolved this issue (Figure S7). FDR‐adjustment was applied to account for multiple comparisons: FDR adjusted *q* < 0.05 was considered as epigenome‐wide significant, while *p* < 1E‐05 was considered to indicate nominal significance.

We looked up CpGs showing associations with physical activity components at a nominally significant level using the EWAS Catalog (http://ewascatalog.org/, downloaded on 16.02.2023) and EWAS Atlas (https://ngdc.cncb.ac.cn/ewas/atlas, downloaded on 16.02.2023). We also performed a look‐up of known associations of the mapped gene for each CpG in previously published GWAS using the GWAS catalog (https://www.ebi.ac.uk/gwas/, downloaded on 16.02.2023). We conducted further gene set enrichment analysis with the WebGestalt (Liao et al., 2019) and ClusterProfiler (Wu et al., 2021). We summarized the results of the latter using the rrvgo R package (Sayols, 2020). We examined whether there were methylation quantitative trait loci (mQTLs) for the CpGs that were associated with MVPA and MET‐Hours using mQTLdb (Gaunt et al., 2016).

## AUTHOR CONTRIBUTIONS

**Fabienne A. U. Fox:** Conceptualization, Methodology, Formal Analysis, Writing—Original Draft Preparation, Visualization; **Dan Liu:** Conceptualization, Methodology, Formal Analysis, Writing—Reviewing and Editing, Visualization; **Monique M. B. Breteler**: Conceptualization, Methodology, Resources, Writing—Reviewing and Editing, Supervision, Data Curation, Funding Acquisition; **N. Ahmad Aziz:** Conceptualization, Methodology, Supervision, Writing—Reviewing and Editing.

## CONFLICT OF INTEREST STATEMENT

The authors report no competing interests.

## Supporting information


**Data S1:** Supporting Information

## Data Availability

The Rhineland Study's dataset is not publicly available because of data protection regulations. Access to data can be provided to scientists in accordance with the Rhineland Study's Data Use and Access Policy. Requests for further information or to access the Rhineland Study's dataset should be directed to rs-duac@dzne.de.
