# Supplementary material for: Physical activity is associated with slower epigenetic ageing—Findings from the Rhineland study
Source: Aging Cell. 2023 Apr 10;22(6):e13828. doi: 10.1111/acel.13828 (PMC10265180; doi:10.1111/acel.13828)
Supplement: Supplementary file 1 — Data S1: Supporting Information [file ACEL-22-e13828-s001.docx]

**Table S1.** Sex-stratified sample demographics.

|  |  |  | Women |  |  |  |  |  | Men |  |  |  | Overall women | Overall  men |  |
| --- | --- | --- | --- | --- | --- | --- | --- | --- | --- | --- | --- | --- | --- | --- | --- |
|  | 30-39 years | 40-49 years | 50-59 years | 60-69 years | 70+ years |  | 30-39 years | 40-49 years | 50-59 years | 60-69 years | 70+ years | p-value |  |  | p-value |
|  | n = 308 | n = 363 | n = 550 | n = 412 | n = 322 | p-value | n = 286 | n = 247 | n = 416 | n = 335 | n = 328 |  | n = 1955 | n = 1612 |  |
| Age (years), mean (SD) | 34.6 (2.8) | 44.9 (2.9) | 54.5 (2.9) | 64.4 (2.9) | 76.1 (4.4) | <0.001 | 34.7 (2.8) | 44.9 (3.2) | 54.3 (2.9) | 64.3 (2.8) | 76.5 (4.7) | <0.001 | 55.2 (13.7) | 56.0 (14.5) | 0.104 |
| Body-mass index (kg/m^2^), mean (SD) | 24.17  (4.22) | 25.02  (5.11) | 25.42  (4.92) | 25.98  (4.66) | 25.67  (3.88) | <0.001 | 25.39  (3.88) | 25.97  (3.72) | 26.98  (4.14) | 27.22  (3.98) | 26.56  (3.38) | <0.001 | 25.31  (4.67) | 26.51  (3.90) | <0.001 |
| Waist-to-hip ratio, mean (SD) | 0.76 (0.07) | 0.78 (0.06) | 0.81 (0.07) | 0.84 (0.07) | 0.86 (0.07) | <0.001 | 0.87 (0.06) | 0.91 (0.06) | 0.95 (0.07) | 0.97 (0.07) | 0.99 (0.07) | <0.001 | 0.81 (0.08) | 0.94 (0.08) | <0.001 |
| Cardiovascular Event, n (% Yes) | 3 (1.0) | 8 (2.2) | 15 (2.7) | 41 (10.0) | 59 (18.3) | <0.001 | 2 (0.7) | 6 (2.4) | 30 (7.2) | 43 (12.8) | 112 (34.2) | <0.001 | 126 (6.5) | 193 (12.0) | <0.001 |
| Smoking, n (% Yes) | 42 (13.6) | 44 (12.1) | 71 (12.9) | 54 (13.1) | 18 (5.6) | 0.006 | 52 (18.2) | 35 (14.2) | 68 (16.4) | 44 (13.1) | 20 (6.1) | <0.001 | 229 (11.7) | 219 (13.6) | 0.103 |
| Diabetes, n (% Yes) | 2 (0.7) | 3 (0.8) | 16 (2.9) | 26 (6.4) | 27 (8.5) | <0.001 | 3 (1.1) | 7 (2.8) | 21 (5.1) | 35 (10.5) | 52 (16.1) | <0.001 | 74 (3.8) | 118 (7.4) | <0.001 |
| Hypertension, n (%) |  |  |  |  |  | <0.001 |  |  |  |  |  | <0.001 |  |  | <0.001 |
| No | 290 (96.0) | 316 (88.0) | 395 (73.7) | 203 (49.5) | 72 (22.6) |  | 255 (89.8) | 197 (79.8) | 234 (56.5) | 136 (41.1) | 73 (22.3) |  | 1276 (66.3) | 895 (55.8) |  |
| Yes, controlled | 5 (1.7) | 17 (4.7) | 75 (14.0) | 111 (27.1) | 96 (30.1) |  | 8 (2.8) | 22 (8.9) | 70 (16.9) | 91 (27.5) | 118 (36.1) |  | 304 (15.8) | 309 (19.3) |  |
| Yes, uncontrolled | 7 (2.3) | 25 (7.0) | 61 (11.4) | 85 (20.7) | 143 (44.8) |  | 19 (6.7) | 27 (10.9) | 108 (26.1) | 100 (30.2) | 124 (37.9) |  | 321 (16.7) | 378 (23.6) |  |
| Yes, unknown | 0 (0.0) | 1 (0.3) | 5 (0.9) | 11 (2.7) | 8 (2.5) |  | 2 (0.7) | 1 (0.4) | 2 (0.5) | 4 (1.2) | 12 (3.7) |  | 25 (1.3) | 21 (1.3) |  |
| Education ISCED11, n (%) |  |  |  |  |  | <0.001 |  |  |  |  |  | 0.031 |  |  | <0.001 |
| high | 195 (63.3) | 215 (59.2) | 269 (48.9) | 152 (36.9) | 75 (23.3) |  | 181 (63.3) | 155 (62.8) | 246 (59.1) | 209 (62.4) | 170 (51.8) |  | 906 (46.3) | 961 (59.6) |  |
| middle | 111 (36.0) | 144 (39.7) | 274 (49.8) | 247 (60.0) | 218 (67.7) |  | 103 (36.0) | 87 (35.2) | 167 (40.1) | 122 (36.4) | 156 (47.6) |  | 994 (50.8) | 635 (39.4) |  |
| low | 2 (0.7) | 4 (1.1) | 7 (1.3) | 13 (3.2) | 29 (9.0) |  | 2 (0.7) | 5 (2.0) | 3 (0.7) | 4 (1.2) | 2 (0.6) |  | 55 (2.8) | 16 (1.0) |  |
| Actimetry season, n (%) |  |  |  |  |  | 0.580 |  |  |  |  |  | 0.554 |  |  | 0.243 |
| spring | 67 (21.75) | 77 (21.21) | 113 (20.55) | 83 (20.15) | 63 (19.57) |  | 68 (23.78) | 55 (22.27) | 87 (20.91) | 81 (24.18) | 58 (17.68) |  | 403 (20.61) | 349 (21.65) |  |
| summer | 69 (22.40) | 74 (20.39) | 127 (23.09) | 105 (25.49) | 81 (25.16) |  | 65 (22.73) | 59 (23.89) | 101 (24.28) | 67 (20.00) | 75 (22.87) |  | 456 (23.32) | 367 (22.77) |  |
| autumn | 77 (25.00) | 112 (30.85) | 164 (29.82) | 126 (30.58) | 101 (31.37) |  | 65 (22.73) | 62 (25.10) | 118 (28.37) | 94 (28.06) | 97 (29.57) |  | 580 (29.67) | 436 (27.05) |  |
| winter | 95 (30.84) | 100 (27.55) | 146 (26.55) | 98 (23.79) | 77 (23.91) |  | 88 (30.77) | 71 (28.74) | 110 (26.44) | 93 (27.76) | 98 (29.88) |  | 516 (26.39) | 460 (28.54) |  |
| Daily Sensor Hours Worn (hours), mean (SD) | 23.83  (0.45) | 23.79  (0.52) | 23.88  (0.39) | 23.78  (0.55) | 23.83  (0.44) | 0.013 | 23.75  (0.67) | 23.85  (0.43) | 23.77  (0.62) | 23.85  (0.46) | 23.77  (0.51) | 0.077 | 23.83  (0.47) | 23.80  (0.55) | 0.083 |
| Daily Energy Expenditure (MET-Hours), mean (SD) | 34.16  (1.32) | 34.24  (1.29) | 34.28  (1.28) | 34.04  (1.33) | 33.51  (1.25) | <0.001 | 33.89  (1.38) | 34.14  (1.37) | 34.11  (1.44) | 33.83  (1.25) | 33.35  (1.46) | <0.001 | 34.07  (1.32) | 33.86  (1.41) | <0.001 |
| Daily Step Count, mean (SD) | 9160.56 (3040.81) | 9369.03 (2985.37) | 9310.67 (3070.42) | 8855.62 (3156.06) | 7370.79 (2877.65) | <0.001 | 8863.77 (3189.72) | 9307.32 (3347.33) | 9352.24 (3529.75) | 8517.31 (2951.95) | 7513.33 (3442.58) | <0.001 | 8882.45 (3113.30) | 8711.01 (3375.99) | 0.115 |
| % Daily Light Intensity Physical Activity, mean (SD) | 22.17  (5.86) | 23.26  (5.92) | 22.75  (5.89) | 22.71  (6.26) | 22.98  (6.08) | 0.203 | 19.20  (5.61) | 20.26  (6.24) | 19.79  (5.65) | 19.34  (5.16) | 18.57  (5.63) | 0.005 | 22.78  (6.01) | 19.42  (5.66) | <0.001 |
| % Daily Moderate-to-Vigorous Physical Activity, mean (SD) | 4.87  (1.66) | 4.96  (1.63) | 4.91  (1.68) | 4.72  (1.76) | 3.89  (1.62) | <0.001 | 4.79  (1.77) | 4.96  (1.77) | 5.05  (1.94) | 4.65  (1.70) | 4.06  (1.97) | <0.001 | 4.71  (1.72) | 4.70  (1.88) | 0.971 |
| % Daily Sedentary, mean (SD) | 72.96  (6.52) | 71.78  (6.52) | 72.34  (6.51) | 72.56  (6.84) | 73.14  (6.61) | 0.058 | 76.01  (6.38) | 74.78  (6.83) | 75.16  (6.26) | 76.01  (5.84) | 77.37  (6.37) | <0.001 | 72.51  (6.63) | 75.88  (6.36) | <0.001 |
| Hannum’s Age acceleration, mean (SD) | -1.05  (5.47) | 0.10  (5.56) | -0.28  (5.80) | -1.15  (5.46) | -0.96  (5.87) | 0.006 | 0.64  (5.43) | 1.68  (5.43) | 2.01  (5.29) | 1.91  (5.40) | 0.78  (5.76) | 0.001 | -0.62  (5.66) | 1.45  (5.48) | <0.001 |
| Horvath’s Age acceleration, mean (SD) | -0.87  (4.81) | 0.05  (5.21) | -0.12  (5.03) | -0.25  (5.31) | -0.69  (5.38) | 0.099 | 0.51  (4.93) | 1.08  (5.80) | 1.55  (4.89) | 0.77  (5.43) | 0.30  (5.80) | 0.016 | -0.33  (5.15) | 0.88  (5.36) | <0.001 |
| PhenoAge acceleration, mean (SD) | -0.79  (6.26) | 0.78  (6.64) | -0.56  (6.71) | -0.49  (7.16) | -0.94  (6.90) | 0.006 | 0.22  (5.75) | 0.59  (6.59) | 1.49  (6.41) | 1.18  (6.35) | 0.49  (6.44) | 0.050 | -0.40  (6.78) | 0.86  (6.33) | <0.001 |
| GrimAge acceleration, mean (SD) | -1.87  (8.31) | -1.64  (5.68) | -1.04  (6.68) | -0.58  (6.97) | 0.33  (7.99) | <0.001 | -0.11  (7.97) | 0.03  (6.18) | 1.20  (6.69) | 1.23  (7.79) | 3.08  (8.64) | <0.001 | -0.96  (7.11) | 1.18  (7.59) | <0.001 |
| % Basophils, mean (SD) | 0.71 (0.78) | 0.79 (0.72) | 0.82 (0.77) | 0.84 (0.82) | 0.84 (0.81) | 0.176 | 0.70 (0.68) | 0.76 (0.68) | 0.71 (0.71) | 0.76 (0.73) | 0.77 (1.29) | 0.776 | 0.81 (0.78) | 0.74 (0.85) | 0.011 |
| % Eosophils, mean (SD) | 2.32 (1.89) | 2.21 (1.62) | 2.68 (1.81) | 2.31 (1.65) | 2.20 (1.87) | <0.001 | 2.86 (1.82) | 2.80 (2.00) | 2.66 (1.93) | 2.48 (1.91) | 1.89 (1.48) | <0.001 | 2.38 (1.77) | 2.52 (1.87) | 0.019 |
| % Neutrophils, mean (SD) | 46.37 (12.41) | 50.11 (11.92) | 47.21 (10.94) | 47.99 (11.97) | 52.91 (11.88) | <0.001 | 48.61 (11.42) | 49.78 (11.27) | 51.57 (10.19) | 53.01 (10.57) | 55.50 (11.09) | <0.001 | 48.72 (11.93) | 51.87 (11.09) | <0.001 |
| % Monophils, mean (SD) | 8.72 (2.23) | 8.66 (2.26) | 8.72 (2.06) | 8.87 (2.18) | 8.94 (2.22) | 0.378 | 8.96 (2.16) | 8.94 (2.09) | 9.08 (2.09) | 9.25 (2.16) | 9.59 (2.48) | 0.001 | 8.77 (2.18) | 9.18 (2.21) | <0.001 |
| % Naïve B cells, mean (SD) | 3.33 (1.59) | 3.05 (1.41) | 3.44 (1.74) | 3.36 (1.79) | 2.80 (1.98) | <0.001 | 3.28 (1.57) | 3.12 (1.48) | 2.91 (1.63) | 2.55 (1.44) | 2.11 (2.12) | <0.001 | 3.23 (1.73) | 2.77 (1.72) | <0.001 |
| % Memory B cells, mean (SD) | 1.88 (0.84) | 1.92 (0.84) | 1.94 (0.87) | 2.37 (5.41) | 2.23 (5.46) | 0.170 | 1.85 (0.93) | 1.98 (1.06) | 1.91 (1.34) | 2.12 (3.63) | 2.28 (4.71) | 0.291 | 2.07 (3.40) | 2.03 (2.84) | 0.730 |
| % Naïve CD4 T cells, mean (SD) | 9.71  (4.44) | 7.92  (4.22) | 8.57  (4.77) | 6.90  (4.32) | 5.40  (3.95) | <0.001 | 7.28  (3.43) | 6.26  (3.61) | 5.93  (3.47) | 5.05  (3.43) | 3.60  (3.15) | <0.001 | 7.75  (4.60) | 5.56  (3.62) | <0.001 |
| % Memory CD4 T cells, mean (SD) | 10.91  (3.72) | 11.34  (3.64) | 12.77  (3.97) | 12.84  (4.39) | 11.44  (4.25) | <0.001 | 9.19  (3.49) | 9.94  (3.49) | 10.52  (3.64) | 10.85  (3.58) | 9.73  (3.67) | <0.001 | 12.01  (4.09) | 10.10  (3.63) | <0.001 |
| % Regulatory T cells, mean (SD) | 0.07  (0.26) | 0.07  (0.23) | 0.07  (0.28) | 0.05  (0.28) | 0.10  (0.36) | 0.268 | 0.09  (0.26) | 0.10  (0.32) | 0.09  (0.30) | 0.07  (0.25) | 0.12  (0.54) | 0.419 | 0.07  (0.28) | 0.09  (0.35) | 0.060 |
| % Naïve CD8 T cells, mean (SD) | 4.35  (2.68) | 3.02  (2.09) | 2.50  (1.90) | 1.80  (1.65) | 1.06  (1.23) | <0.001 | 3.62  (2.20) | 2.61  (2.04) | 1.78  (1.57) | 1.27  (1.21) | 0.92  (1.09) | <0.001 | 2.50  (2.19) | 1.95  (1.89) | <0.001 |
| % Memory CD8 T cells, mean (SD) | 6.81  (4.76) | 6.16  (4.86) | 6.07  (5.04) | 7.38  (6.27) | 6.48  (6.18) | 0.003 | 7.45  (5.05) | 7.47  (5.54) | 7.10  (5.88) | 6.88  (5.84) | 7.26  (6.99) | 0.709 | 6.54  (5.46) | 7.21  (5.93) | 0.001 |
| % Natural killer cells, mean (SD) | 0.71 (0.78) | 0.79 (0.72) | 0.82 (0.77) | 0.84 (0.82) | 0.84 (0.81) | 0.020 | 0.70 (0.68) | 0.76 (0.68) | 0.71 (0.71) | 0.76 (0.73) | 0.77 (1.29) | <0.001 | 5.39 (2.42) | 6.02 (2.73) | <0.001 |
| Systolic blood pressure (mm Hg), mean (SD) | 113.50  (9.63) | 115.87 (12.21) | 121.01 (13.73) | 128.13 (15.88) | 138.60 (18.09) | <0.001 | 125.13  (8.81) | 126.47 (11.34) | 130.63 (13.21) | 131.64 (14.73) | 137.55 (18.46) | <0.001 | 123.25  (16.49) | 130.63  (14.50) | <0.001 |
| Diastolic blood pressure (mm Hg), mean (SD) | 70.05  (8.38) | 72.99  (9.33) | 74.97  (8.45) | 74.63  (8.88) | 74.36  (10.12) | <0.001 | 74.28  (7.35) | 77.14  (8.87) | 80.42  (9.34) | 78.41  (9.57) | 75.28  (10.25) | <0.001 | 73.65  (9.14) | 77.37  (9.46) | <0.001 |
| Cholesterol (mg/dL), mean (SD) | 174.85 (31.27) | 186.55 (33.62) | 209.48 (36.27) | 219.34 (40.37) | 215.10 (40.39) | <0.001 | 180.83 (36.32) | 194.32 (36.17) | 202.46 (38.60) | 196.66 (39.04) | 187.14 (38.47) | <0.001 | 202.59  (40.12) | 193.05  (38.65) | <0.001 |
| High-density lipoprotein (mg/dL), mean (SD) | 66.53  (15.01) | 66.32  (15.62) | 71.23  (17.61) | 71.75  (18.02) | 72.10  (17.40) | <0.001 | 50.69  (12.22) | 52.69  (12.89) | 51.70  (14.37) | 54.13  (13.49) | 57.00  (14.84) | <0.001 | 69.80  (17.08) | 53.24  (13.86) | <0.001 |
| Low-density lipoprotein (mg/dL), mean (SD) | 103.32 (27.11) | 114.69 (31.86) | 131.60 (34.71) | 139.45 (39.38) | 133.57 (38.10) | <0.001 | 118.72 (33.30) | 130.66 (33.11) | 135.55 (34.75) | 129.82 (35.42) | 119.07 (35.51) | <0.001 | 125.85  (36.96) | 127.27  (35.19) | 0.250 |
| Triglycerides (mg/dL), mean (SD) | 82.08  (41.15) | 85.08  (40.02) | 98.84  (52.40) | 107.55 (57.10) | 109.82 (45.56) | <0.001 | 122.19 (104.24) | 116.19 (61.20) | 143.14 (99.66) | 132.37 (73.36) | 118.33 (63.09) | <0.001 | 97.16  (49.72) | 128.11  (84.41) | <0.001 |
| Insulin (mU/L), mean(SD) | 8.97 (5.31) | 8.75 (5.18) | 8.93 (6.05) | 9.88 (6.47) | 10.72 (5.75) | <0.001 | 10.55 (7.60) | 10.33 (8.85) | 12.75 (11.15) | 12.16 (8.53) | 11.91 (6.98) | 0.002 | 9.39 (5.86) | 11.70 (8.95) | <0.001 |
| **Abbreviations:** Metabolic-Equivalent Hours (MET-Hours), Standard Deviation (SD), 95% Confidence Interval (95% CI) | | | | | | | | | | | | | | | |

**Table S2.** Direct and indirect effects of physical activity on cardiovascular disease risk mediated by epigenetic ageing (GrimAge acceleration), while keeping the covariates at a constant.

|  |  | **Indirect effect** | | | **Direct effect** | | | **Total effect** | | | **Mediated effect** | | |
| --- | --- | --- | --- | --- | --- | --- | --- | --- | --- | --- | --- | --- | --- |
| **Outcome** | **Predictor** | **-1 SD**  **[95% CI]** | **Average**  **[95% CI]** | **+1 SD**  **[95% CI]** | **-1SD**  **[95% CI]** | **Average**  **[95% CI]** | **+1 SD**  **[95% CI]** | **-1 SD**  **[95% CI]** | **Average**  **[95% CI]** | **+1 SD**  **[95% CI]** | **-1 SD** | **Average** | **+1 SD** |
| FAMD cardiovascular disease component 1 | MET-Hours | -0.0185*  [-0.0379;  -0.0055] | -0.0085*  [-0.0191;  -0.0026] | 0.0015  [-0.0044;  0.0085] | NA^a^ | -0.5068***  [-0.6118;  -0.4139] | NA^a^ | -0.5254***  [-0.6291;  -0.4305] | -0.5154***  [-0.6198;  -0.4230] | -0.5054***  [-0.6121;  -0.4149] | 3.53% | 1.66% | NA^b^ |
|  | Step Count | -0.0215*  [-0.0451;  -0.0066] | -0.0108*  [-0.0224;  -0.0032] | -0.0001  [-0.0068;  0.0052] | NA^a^ | -0.4900***  [-0.6002;  -0.3981] | NA^a^ | -0.5116***  [-0.6227;  -0.4190] | -0.5009***  [-0.6098;  -0.4071] | -0.4901***  [-0.6012;  -0.3988] | 4.21% | 2.16% | 0.02% |
|  | % MVPA | -0.0204*  [-0.0428;  -0.0062] | -0.0099*  [-0.0209;  -0.0028] | 0.0006  [-0.0057;  0.0066] | NA^a^ | -0.4381***  [-0.5513;  -0.3456] | NA^a^ | -0.4585***  [-0.5724;  -0.3647] | -0.4480***  [-0.5632;  -0.3553] | -0.4376***  [-0.5493;  -0.3422] | 4.44% | 2.21% | NA^b^ |
| FAMD cardiovascular disease component 2 | MET-Hours | 0.0070  [-0.0010;  0.0201] | 0.0032  [-0.0005;  0.0096] | -0.0005  [-0.0055;  0.0014] | 0.3394***  [0.2040;  0.4762] | 0.2235***  [0.1552;  0.3030] | 0.1076*  [0.0211;  0.1966] | 0.3463***  [0.2164;  0.4862] | 0.2267***  [0.1588;  0.3064] | 0.1071*  [0.0218;  0.1966] | 2.01% | 1.42% | NA^b^ |
|  | Step Count | 0.0080  [-0.0015;  0.0220] | 0.0040  [-0.0009;  0.0115] | 0.0001  [-0.0021;  0.0030] | 0.3273***  [0.1878;  0.4693] | 0.2306***  [0.1489;  0.3158] | 0.1339***  [0.0583;  0.2168] | 0.3353***  [0.1973;  0.4762] | 0.2346***  [0.1572;  0.3180] | 0.1340***  [0.0579;  0.2153] | 2.39% | 1.72% | 0.02% |
|  | % MVPA | 0.0084  [-0.0004;  0.0218] | 0.0041  [-0.0002;  0.0111] | -0.0002  [-0.0034;  0.0023] | NA^a^ | 0.1746***  [0.1085;  0.2497] | NA^a^ | 0.1830***  [0.1125;  0.2563] | 0.1787***  [0.1111;  0.2521] | 0.1744***  [0.1083;  0.2488] | 4.58% | 2.28% | NA^b^ |
| Framingham Heart Study cardiovascular score | MET-Hours | -0.0016**  [-0.0032;  -0.0006] | -0.0009**  [-0.0017;  -0.0003] | -0.0002  [-0.0007;  0.0003] | -0.0181***  [-0.0263;  -0.0096] | -0.0123***  [-0.0164;  -0.0080] | -0.0065***  [-0.0101;  -0.0029] | -0.0197***  [-0.0278;  -0.0112] | -0.0132***  [-0.0173;  -0.0090] | -0.0066***  [-0.0103;  -0.0032] | 8.30% | 6.83% | 2.46% |
|  | Step Count | -0.0018**  [-0.0032;  -0.0008] | -0.0010**  [-0.0018;  -0.0004] | -0.0002  [-0.0007;  0.0002] | -0.0167***  [-0.0240;  -0.0087] | -0.0118***  [-0.016;  -0.0078] | -0.0069***  [-0.0104;  -0.0038] | -0.0184***  [-0.0261;  -0.0107] | -0.0128***  [-0.0168;  -0.0088] | -0.0072***  [-0.0106;  -0.0040] | 9.62% | 7.82% | 3.19% |
|  | % MVPA | -0.0017**  [-0.0031;  -0.0006] | -0.0009**  [-0.0017;  -0.0003] | -0.0002  [-0.0007;  0.0003] | -0.0153***  [-0.0229;  -0.0079] | -0.0111***  [-0.0154;  -0.0071] | -0.0069***  [-0.0104;  -0.0038] | -0.0170***  [-0.0248;  -0.0092] | -0.0120***  [-0.0162;  -0.0080] | -0.0071***  [-0.0106;  -0.0039] | 9.76% | 7.70% | 2.74% |
| ESC SCORE2 | MET-Hours | -0.0006***  [-0.0010;  -0.0003] | -0.0003**  [-0.0005;  -0.0001] | 0.0001  [-0.0001;  0.0003] | -0.0060***  [-0.0083;  -0.0037] | -0.0044***  [-0.0055;  -0.0031] | -0.0028***  [-0.0037;  -0.0017] | -0.0065***  [-0.0088;  -0.0042] | -0.0046***  [-0.0058;  -0.0034] | -0.0027***  [-0.0037;  -0.0017] | 8.49% | 5.49% | NA^b^ |
|  | Step Count | -0.0006***  [-0.0011;  -0.0003] | -0.0003***  [-0.0005;  -0.0002] | 0.0001  [-0.0002;  0.0002] | -0.0065***  [-0.0090;  -0.0041] | -0.0046***  [-0.0059;  -0.0033] | -0.0027***  [-0.0037;  -0.0017] | -0.0072***  [-0.0095;  -0.0049] | -0.0049***  [-0.0062;  -0.0036] | -0.0027***  [-0.0036;  -0.0017] | 8.87% | 6.44% | NA^b^ |
|  | % MVPA | -0.0006***  [-0.0010;  -0.0003] | -0.0003**  [-0.0005;  -0.0001] | 0.0001  [-0.0001;  0.0002] | -0.0060***  [-0.0085;  -0.0037] | -0.0042***  [-0.0055;  -0.0029] | -0.0025***  [-0.0034;  -0.0014] | -0.0066***  [-0.0089;  -0.0042] | -0.0045***  [-0.0057;  -0.0032] | -0.0024***  [-0.0033;  -0.0013] | 8.91% | 6.29% | NA^b^ |
| ASCVD Score | MET-Hours | -0.0004  [-0.0010;  -0.0001] | -0.0002  [-0.0004;  0.0001] | 0.0001  [-0.0001;  0.0003] | NA | -0.0054***  [-0.0077;  -0.0033] | NA | -0.0059***  [-0.0081;  -0.0036] | -0.0056***  [-0.0079;  -0.0035] | -0.0054***  [-0.0077;  -0.0032] | 7.30% | 3.42% | NA^b^ |
|  | Step Count | -0.0005*  [-0.0010;  -0.0001] | -0.0002  [-0.0005;  0.0001] | 0.0001  [-0.0001;  0.0002] | -0.0114***  [-0.0165;  -0.0064] | -0.0078***  [-0.0109;  -0.0049] | -0.0043***  [-0.0061;  -0.0024] | -0.0118***  [-0.0170;  -0.0068] | -0.0080***  [-0.0111;  -0.0051] | -0.0043***  [-0.0062;  -0.0023] | 3.86% | 2.80% | NA^b^ |
|  | % MVPA | -0.0005*  [-0.0010;  -0.0001] | -0.0002  [-0.0005;  0.0001] | 0.0001  [-0.0001;  0.0002] | NA | -0.0057***  [-0.0080;  -0.0035] | NA | -0.0062***  [-0.0085;  -0.0039] | -0.0059***  [-0.0082;  -0.0037] | -0.0057***  [-0.008;  -0.0035] | 7.39% | 3.67% | NA^b^ |
| Significance levels: *) p ≤ 0.05, **) p ≤ 0.01, ***) p ≤ 0.001.  ^a^) Models with only linear effect.  ^b^) Models with opposite direct and indirect effects.  **Abbreviations:** Factor Analysis for Mixed Data (FAMD), Metabolic-Equivalent Hours (MET-Hours), %Moderate-to-Vigorous Physical Activity (MVPA), Standard Deviation (SD), 95% Confidence Interval (95% CI) | | | | | | | | | | | | | |

**Table S3.** Mediation effect of olfactory performance (negative control variable) on the association between physical activity and epigenetic ageing.

|  |  | **Indirect effect** | | | **Direct effect** | | | **Total effect** | | | **Percentage mediated** | | |  |
| --- | --- | --- | --- | --- | --- | --- | --- | --- | --- | --- | --- | --- | --- | --- |
| **Mediator** | **Predictor** | **-1 SD**  **[95% CI]** | **Average**  **[95% CI]** | **+1 SD**  **[95% CI]** | **-1SD**  **[95% CI]** | **Average**  **[95% CI]** | **+1 SD**  **[95% CI]** | **-1 SD**  **[95% CI]** | **Average**  **[95% CI]** | **+1 SD**  **[95% CI]** | **-1 SD** | **Average** | **+1 SD** |  |
| Olfactory performance | MET-Hours | NA^a^ | 0.0004  [-0.0008;  0.0025] | NA^a^ | -0.1310***  [-0.2027;  -0.0606] | -0.0584**  [-0.0960;  -0.0240] | 0.0142  [-0.0333;  0.0587] | -0.1306  [-0.2022;  -0.0591] | -0.0580  [-0.0958;  -0.0230] | 0.0146  [-0.0333;  0.0591] | NA^b^ | NA^b^ | 2.61% |  |
|  | Step Count | NA^a^ | 0.0003  [-0.0009;  0.0023] | NA^a^ | -0.1555***  [-0.2331;  -0.0823] | -0.0760***  [-0.1186;  -0.0418] | 0.0036  [-0.0398;  0.0416] | -0.1552  [-0.2322;  -0.0817] | -0.0756  [-0.1181;  -0.0408] | 0.0040  [-0.0385;  0.0424] | NA^b^ | NA^b^ | 8.21% |  |
|  | % MVPA | NA^a^ | 0.0001  [-0.0012;  0.0019] | NA^a^ | -0.1416***  [-0.2167;  -0.0679] | -0.0664***  [-0.1070;  -0.0305] | 0.0088  [-0.0360;  0.0493] | -0.1415  [-0.2169;  -0.0672] | -0.0663  [-0.1062;  -0.0296] | 0.0089  [-0.0359;  0.0495] | NA^b^ | NA^b^ | 1.43% |  |
| Significance levels: *) p ≤ 0.05, **) p ≤ 0.01, ***) p ≤ 0.001.  ^a^) Models with only linear effect.  ^b^) Models with opposite direct and indirect effects.  Abbreviations: Metabolic-Equivalent Hours (MET-Hours), Moderate-to-Vigorous Physical Activity (MVPA), Standard Deviation (SD), 95% Confidence Interval (95% CI) | | | | | | | | | | | | | | |

**Table S4.** EWAS results of physical activity with lookup results of other EWAS and GWAS trait associations.

**a CpGs showing associations with %MVPA at a nomonially significant level**

| **cpg** | **chr** | **pos** | **Nearest Gene** | **beta** | **Standard error** | **P-value** | **fdr** | **mQTL** | **Other EWAS trait associations** | **Other GWAS trait association** |
| --- | --- | --- | --- | --- | --- | --- | --- | --- | --- | --- |
| cg27071152 | chr7 | 39649443 | LOC646999 | -0.489 | 0.097 | 4.459e-07 | 0.252 | rs34516389, rs61493712, rs4723882, rs4723883, rs4723884, rs4723885, rs6968742, rs2329460, rs2329461, rs2329462, rs7796063, rs2329463, rs10486798, rs2329459, rs10486800, rs77757397, rs10236547, rs7777173, rs10258946, rs1989533, rs4723881, rs10245247, rs10266149, rs7801411, rs10270753, rs6462944, rs4451213, rs112444584, rs17416652, rs17507685, rs17507748, rs144904297, rs7807233, rs77032006, rs75249621, rs77946796, rs76408259, rs75432578, rs117810342, rs17508696, rs74491221, rs78407061, rs77470298, rs74352433, rs7810999, rs142376052, rs142637514, rs11769907, rs7777339, rs58299139, rs7785964, rs2329455, rs17171603, rs149006472, rs73375695, rs17620556, rs118177415, rs141548457, rs17687994, rs140385842, rs73130994, rs192513129, rs11976477, rs6462951, rs117798313, rs138896627, rs75951304, rs10228362, rs10252318, rs117120624, rs4394293, rs78858520, rs192807164, rs11765134, rs79032725, esv2666474, esv2662059, rs78689737, rs148402812, rs147817677, rs11764316, rs117092168, rs112320570, rs74456323, rs77785607, rs75559609, rs112794127, rs73132703, rs2329400, rs184882279, rs147231995, rs140611016, rs2108079, rs77716972, rs77908445, rs77229349, rs4273762, rs2876841, rs2876842, rs17687569, rs117693276, rs4720329, rs6958662, rs7809088, rs4469364, rs6963017, rs150928593, rs202145359 | Age, clear cell renal carcinoma, tissue |  |
| cg00484396 | chr16 | 3507460 | NAA60 | 0.288 | 0.058 | 6.740e-07 | 0.352 | rs250626, rs37774, rs37834, rs1690446, rs1690447, rs1690449, rs1690450, rs4786421, rs166103, rs1137454, rs1617722, rs250631, rs37838, rs200750285, rs59698966, rs6501177, rs250630, rs2087997, rs250629, rs40447, rs1635392, rs37835, rs17525862, rs37772, rs250628, rs6501176, rs37766, rs28810, rs40535, rs40363, rs39730, rs250632, rs1690444, rs40223, rs17554547, rs192228, rs37837, rs37839, rs250636, rs250637, rs37771, rs37769, rs37768, rs37767, rs28401, rs1690443, rs1635393, rs1690451, rs55989968, rs72778117, rs37773, rs7206156, rs1690441, rs2240075, rs13740, rs757270, rs9932315, rs2285815, rs11077343, rs11077344, rs2379828, rs2890267, rs2379829, rs2379830, rs4010630, rs11077345, rs9926609, rs200796620, rs2526285, rs2526284, rs8061324, rs37824, rs166101, rs250536, rs250533, rs250532, rs250531, rs111894801, rs250526, rs250525, rs185148, rs37821, rs37822, rs37823, rs190374, rs37828, rs250534, rs250530, rs250529, rs171634, rs166100, rs112481124, rs40534, rs37825, rs37826, rs37827, rs42223, rs40222, rs2526286, rs2641789, rs42971, rs57537175, rs57077984, rs2680235, rs35574584, rs250528, rs3816916, rs27242, rs37832, rs34496784, rs4453493, rs7500590, rs4785936, rs4010628, rs72778116, rs250523, rs2736, rs8060367, rs757269, rs2379827, rs2379831, rs9926612, rs1003330, rs2006264, rs7190818, rs4786425, rs3859150, rs4010629, rs2015640, rs12448166, rs9936087, rs11643487, rs27230, rs37810, rs39728, rs17136359, rs112036489, rs9932424, rs7195948, rs9929284, rs37800, rs37816, rs250540, rs2680221, rs37814, rs1134597, rs138180515, rs9635563, rs9635548, rs28621773, rs28735976, rs11077331, rs9931807, rs9938399, rs4785934, rs7404416, rs7404429, rs9921169, rs9924519, rs9922087, rs9302871, rs28089, rs9635547, rs42329, rs9930379, rs9922168, rs27235, rs1149492, rs27241, rs27228, rs35214904, rs147919264, rs8054222, rs55732163, rs7206007, rs10852706, rs7188903, rs35394728, rs1218762, rs1218760, rs11648783, rs4429296, rs4318205, rs1230946, rs67547123, rs35918685, rs199559937, rs11639510, rs34284914, rs28603, rs1129568, rs56107802, rs201965350, rs67218524, rs113977824, rs1635394, rs9635562, rs40633, rs34805102, rs27227, rs27238, rs27239, rs200842336, rs4785933, rs17612463, rs9937267, rs9921152, rs8053830, rs27225, rs27226, rs200180082, rs34370895, rs2522068, rs11077334, rs35693744, rs27231, rs27232, rs27233, rs77759662, rs10048083, rs67712995, rs11077335, rs7194596, rs72778130, rs74497899, rs8061444, rs11077337, rs2270497, rs3848356, rs2270494, rs8056000, rs8046072, rs35984213, rs9790, rs12596890, rs1859249, rs6500549, rs111843862, rs12448573, rs35175748, rs113843744, rs3843728, rs72778138, rs2522069, rs35749441, rs35763431, rs34253072, rs4785932, rs3848355, rs138922864, rs1231099, rs56793723, rs13330501, rs35160989, rs1231090, rs12920536, rs4578649, rs7196196, rs200368877, rs1044390, rs28644313, rs3859143, rs12447807, rs2074366, rs8062722, rs4786405, rs7204571, rs6501171, rs11643983, rs4786412, rs58904842, rs3848358, rs3848357, rs112767262, rs140353974, rs62031767, rs10048148, rs4786416, rs11076782, rs11077330, rs72778152, rs3794702, rs67081976, rs8055172, rs7202449, rs72760803, rs8048452, rs8050376, rs34780561, rs3760083, rs17685105, rs1878931, rs4786406, rs72760809, rs12935546, rs11646775, rs7199019, rs11648068, rs2106862, rs3207360, rs10163302, rs37801, rs62031804, rs11077329, rs11642109, rs4260059, rs7198051, rs200952701, rs201069917, rs72778133, rs116926826, rs1639150, rs13926, rs28460311, rs9930893, rs4260060, rs7191949, rs1859379, rs8044743, rs4785937, rs7202780, rs9925749, rs1024573, rs59830811, rs2890116, rs1609847, rs12925683, rs11643501, rs79684678, rs56171223, rs79318788, rs76907289, rs78876543, rs1968055, rs56078885, rs75917204, rs118185739, rs117189983, rs55813818, rs129987, rs2106863, rs38025, rs56403655, rs78073400, rs77725133, rs12448488, rs11644228, rs73503305, rs7190721, rs7206748, rs67264850, rs145911924, rs3810812, rs7203586, rs11861770, rs7191237, rs3810809, rs9934814, rs17255757, rs72776389, rs149575887, rs72778151, rs75712687, rs6500552, rs75987714, rs11645975, rs72776362, rs11646280, rs8061528 | Tissue, fetal vs adult liver, age 4 vs age 0, age | eosinophil counts, eosinophil percentage of white cells, diastolic blood pressure, systolic blood pressure, mean arterial pressure, sunburns, tuberculosis, cerebral amyloid deposition (pet imaging) |
| cg02024705 | chr4 | 155471894 | PLRG1 | 0.102 | 0.021 | 1.671e-06 | 1 |  |  | serum levels of protein LGALS7, venous thromboembolism, apolipoprotein B levels, ischemic stroke, blood protein levels, serum levels of protein ALDH1A3, cholesteryl esters to total lipids ratio in chylomicrons and extremely large VLDL, total cholesterol levels, low density lipoprotein cholesterol levels, fibrinogen levels or plasminogen activator inhibitor 1 levels (pleiotropy), fibrinogen levels or factor VII levels or factor XI levels or tissue plasminogen activator levels (pleiotropy), fibrinogen levels or tissue plasminogen activator levels (pleiotropy), phenylalanine levels, fibrinogen levels, fibrinogen, chronic obstructive pulmonary disease-related biomarkers, low density lipoprotein cholesterol levels, serum levels of protein SYK |
| cg21164418 | chr4 | 185339813 | IRF2 | -0.107 | 0.023 | 3.808e-06 | 1 |  |  | adolescent idiopathic scoliosis, blood copper measurement, sex hormone-binding globulin measurement, antithrombotic agent use measurement, adverse effect, response to drug, hippocampal volume |
| cg20630690 | chr19 | 10420301 | ZGLP1 | 0.080 | 0.018 | 5.923e-06 | 1 |  | DNA methylation | inflammatory skin disease, COVID-19 (critical illness vs population or mild symptoms), intercellular adhesion molecule 1 levels, low density lipoprotein cholesterol levels, intercellular adhesion molecule 5 levels, white blood cell count, lymphocyte-to-monocyte ratio, lymphocyte count, protein quantitative trait loci, childhood ear infection |
| cg01980521 | chr9 | 110802060 | MUSK | 0.105 | 0.023 | 7.326e-06 | 1 |  |  | body mass index, heel bone mineral density, protein quantitative trait loci (liver), maximum stenosis, orofacial cleft x maternal periconceptional smoking interaction (1df), plasma PCSK9 levels, height, dementia in non-APOE e4 carriers, lung adenocarcinoma, non-small cell lung cancer, heel bone mineral density, cortical thickness (MOSTest), lung function (FVC), plasma factor V levels in venous thrombosis (conditioned on rs6027), mean degree of stenosis, type 2 diabetes (dietary heme iron intake interaction) |
| cg12078958 | chr2 | 76925561 | LRRTM4 | 0.106 | 0.024 | 8.287e-06 | 1 |  | Age, maternal body mass index, nitrogen dioxide exposure, educational attainment | insomnia, ease of getting up in the morning, metabolite levels, number of sexual partners, lung function, gut microbiota (bacterial taxa, hurdle binary method), total brain volume change rate, cortex volume change rate, schizophrenia, DNA methylation variation (age effect), urinary uromodulin levels (indexed to creatinine), executive function (longitudinal)  f-savoury food liking (derived food-liking factor), f-acquired taste liking (derived food-liking factor), f-seafood liking (derived food-liking factor), shellfish liking, venous thromboembolism adjusted for sickle cell variant rs77121243-t, peripheral arterial disease (traffic-related air pollution interaction), adipsin levels, risk-taking behavior (multivariate analysis), indoleacetate levels in elite athletes, morning person, chronotype, carotid plaque maximum area, sum of carotid plaque area, externalizing behaviour (multivariate analysis), educational attainment, smoking initiation, insomnia, pork consumption, oily fish consumption, body size at age 10, mackerel liking, sardines liking, herring liking, f-small fish liking (derived food-liking factor), smoked fish liking, f-oily fish liking (derived food-liking factor), hypotension and cognitive impairment, morningness, carotid intima-media thickness (mean of the maximum cimt), palmitoleoyl-arachidonoyl-glycerol (16:1/20:4) [2] levels in elite athletes, s-7-hydroxywarfarin levels, risk-taking tendency (4-domain principal component model), response to lamotrigine and valproic acid in genetic generalized epilepsy, lip morphology, igg glycosylation, plasma omega-6 polyunsaturated fatty acid levels (linoleic acid), verbal declarative memory, menstruation quality of life impact (stiff neck), COVID-19 (critical illness vs population), baked/steamed fish liking, germline telomere length in neuroblastoma, f-fish liking (derived food-liking factor), time to colon cancer recurrence, rubella, attention deficit hyperactivity disorder symptom score, logical memory (delayed recall) in normal cognition, fasting plasma glucose, drink temperature, depression x vitamin d prs interaction (cojo adjusted), hdl cholesterol levels, total amyloid (snp x snp interaction), igg glycosylation, lung cancer (snp x snp interaction), core binding factor acute myeloid leukemia |

**Abbreviations:** chr (chromosome), pos (position).

**b CpGs showing associations with MET-Hours at a nominally significant level**

| **cpg** | **chr** | **pos** | **Nearest Gene** | **beta** | **Standard error** | **P-value** | **fdr** | **mQTL** | **Other EWAS trait associations** | **Other GWAS trait association** |
| --- | --- | --- | --- | --- | --- | --- | --- | --- | --- | --- |
| cg02493524 | chr5 | 3768103 | *IRX1* | 4.01E-03 | 7.67E-04 | 1.88E-07 | 0.168 |  |  | waist-to-hip ratio, waist-hip index, red cell distribution width, hip index, hip circumference, glomerular filtration rate, vertical cup-disc ratio, medication use (vasodilators used in cardiac diseases), QT interval, rheumatoid factor seropositivity in rheumatoid arthritis, adolescent idiopathic scoliosis, lung function, myocardial infarction, body mass index, human papilloma virus 16 positive oropharyngeal cancer, thrombin-antithrombin complex levels in ischemic stroke, plasma trimethyllysine levels, immune response to smallpox vaccine (IL-6), placental abruption |
| cg12325351 | chr8 | 48691000 | *PRKDC* | -1.07E-03 | 2.13E-04 | 6.04E-07 | 0.291 |  |  | eosinophil counts, monocyte count, basophil count, hemoglobin, neutrophil count, mean corpuscular hemoglobin, monocyte percentage of white cells, neutrophil percentage of white cells, eosinophil percentage of white cells, adult body size, lobe attachment, mean corpuscular volume, externalizing behaviour, age at menopause, neutrophil percentage of granulocytes, eosinophil percentage of granulocytes, eosinophil basophil counts, granulocyte percentage of myeloid white cells, lymphocyte percentage of white cells, insomnia, basophil percentage of white cells |
| cg21496511 | chr17 | 56232384 | *OR4D1* | -1.41E-03 | 2.86E-04 | 8.54E-07 | 0.348 |  |  | blood protein levels, serum levels of protein RAB26, estrone/androstenedione ratio in resected early stage-receptor positive breast cancer, educational attainment, Ischemic stroke (cardioembolic) |
| cg18193094 | chr6 | 101846905 | *GRIK2* | 2.06E-03 | 4.30E-04 | 1.80E-06 | 0.381 |  | ageing, maternal lead exposure, methylation, tissue, Growth differentiation factor 15 | glaucoma, waist circumference, A body shape index, self-reported math ability, CD24 on IgD+ CD24+ B cell, CD24 on memory B cell, cognitive function, alcohol consumption x hours spent using computers, alcohol consumption x hours spent watching television, depression, weight, metabolite levels, adolescent idiopathic scoliosis, serum albumin levels, body mass index, 3-month functional outcome in ischaemic stroke, smoking initiation, disability level, anxiety severity x hours spent watching television, heart rate response to recovery post exercise, colonoscopy-negative controls vs population controls, number of sexual partners, endometriosis, calcium levels, renal function-related traits, biochemical measures, coenzyme Q10 levels, pseudouridine levels, CAG-448 sp000433415 abundance in stool, blood osmolality, neuritic plaques, economic and political preferences, bipolar disorder or major depressive disorder |
| cg19237047 | chr19 | 47748856 | *NOP53* | -1.27E-03 | 2.66E-04 | 2.09E-06 | 0.381 | rs3026889, rs12751705, rs12730017, rs17359650 | tissue | interferon-related traits |
| cg23299462 | chr4 | 78170095 | *CCNG2* | -1.37E-03 | 2.90E-04 | 2.45E-06 | 0.397 |  |  | blood protein levels, neurofibrillary tangles, response to tocilizumab in rheumatoid arthritis, adolescent idiopathic scoliosis, total intracranial volume, protein quantitative trait loci (liver), 3-hydroxypropylmercapturic acid levels in smokers, postprandial glucose in prediabetes, lung function, total amyloid |
| cg08264338 | chr5 | 143303200 | *NR3C1* | -1.89E-03 | 4.04E-04 | 2.96E-06 | 0.450 |  | preterm birth, age, tissue, triglyceride level | hip circumference, atrial fibrillation, carotid intima media thickness, hematocrit, hemoglobin, sex hormone-binding globulin levels, height, testosterone levels, night sleep phenotypes, eosinophil counts, protein quantitative trait loci, neutrophil percentage of white cells, lymphocyte percentage of white cells, waist circumference, appendicular lean mass, Otitis media, breast cancer, eosinophil percentage of white cells, multiple sclerosis, llung function, response to esketamine in treatment resistant depression, migraine, spatial processing, medication use (thyroid preparations), endometriosis |
| cg00056202 | chr8 | 9791350 | *TNKS* | 2.51E-03 | 5.38E-04 | 3.28E-06 | 0.492 |  | cancer, multiple sclerosis, tissue, age | A body shape index, waist circumference, waist-to-hip ratio, waist-hip index, diastolic blood pressure x smoking status, diastolic blood pressure x smoking status, neuroticism, highest math class taken, self-reported math ability, educational attainment, endometriosis or asthma (pleiotropy), systolic blood pressure x smoking status, serum alkaline phosphatase levels, triglyceride levels in VLDL, heel bone mineral density, white matter hyperintensity volume, deep white matter hyperintensities, triglyceride levels, systolic blood pressure, total lipid levels, global cognition, general risk tolerance, systolic blood pressure x alcohol consumption, diastolic blood pressure x alcohol consumption, cortical surface area, aspartate aminotransferase levels, white matter microstructure, estimated glomerular filtration rate, cervical cancer, medication use (antithrombotic agents), multiple myeloma (hyperdiploidy), positive affect, well-being spectrum, free cholesterol levels, phospholipid levels, concentration of VLDL particles, metabolite levels, smoking, fasting leucine, protein quantitative trait loci (liver), pulse pressure x alcohol consumption, schizophrenia, High density lipoprotein cholesterol levels, Mosquito bite size, C-reactive protein levels, triglyceride levels in chylomicrons and VLDL, total lipid levels in chylomicrons and VLDL, concentration of chylomicrons and VLDL particles, metabolic biomarkers, aerodigestive squamous cell cancer (pleiotropy), bone mineral density, adolescent idiopathic scoliosis, LDL cholesterol levels, bipolar disorder, response to radiotherapy in nasopharyngeal carcinoma, corneal endothelial cell size variation, white matter hyperintensity volume x hypertension interaction, regular attendance at a religious group, phospholipid levels in chylomicrons and VLDL, cholesterol levels in VLDL, ratio of polyunsaturated fatty acids to monounsaturated fatty acids, triglycerides to total lipids ratio in LDL, cholesterol levels in chylomicrons VLDL, cholesteryl ester levels in chylomicrons and VLDL, free cholesterol levels in chylomicrons and VLDL, triglycerides to total lipids ratio in HDL, diameter of VLDL particles, epilepsy, age at first birth, body fat percentage, alanine aminotransferase levels, trauma exposure, systemic lupus erythematosus |
| cg17385847 | chr5 | 170815958 | *NPM1* | 1.28E-03 | 2.78E-04 | 4.23E-06 | 0.644 | rs1145335, rs9813835, rs6441402, rs7629279, rs9815749, rs6775789, rs6781584, rs12495397, rs6806786, rs6810101, rs6777763, rs13059686, rs11342443, rs7650690, rs9811878, rs9840505, rs9876383, rs1903744, rs66907338, rs71625567, rs1402252, rs3773922, rs10936343, rs10936345, rs10936346, rs1012364, rs7617184, rs41373752, rs13092027, rs35495062, rs7297, rs9849107, rs10936352, rs1144112, rs1143781, rs7614943, rs1165261, rs7653531, rs6775161, rs16334, rs80236436, rs35226815, rs1165536, rs201890430, rs6784601, rs6793682, rs1829551, rs13098584, rs16962393 | IL-13 treatment, age | self-reported math ability, highest math class taken, height, vertigo, multisite chronic pain, vertex-wise cortical thickness |
| cg09557462 | chr8 | 26721736 | *ADRA1A* | 1.87E-03 | 4.09E-04 | 4.97E-06 | 0.753 | rs876714, rs1877675, rs4066629, rs1871900, rs11985023 | cancer, age, atopy, tissue | PHF-tau, white matter hyperintensities, executive function, global cognition, gut microbiota abundance, response to hydroxyurea, reaction time, bitter alcoholic beverage consumption, height, blood metabolite levels, response to amphetamines, tinnitus in cisplatin-treated testicular cancer |
| cg07093177 | chr12 | 64542573 | *RP11-196H14.3* | -2.65E-03 | 5.85E-04 | 6.06E-06 | 0.808 |  |  |  |
| cg15885440 | chr12 | 29302714 | *FAR2* | 1.61E-03 | 3.62E-04 | 9.72E-06 | 0.808 |  | cancer | platelet count, volume and distribution width, balding type 1, male-pattern baldness, protein quantitative trait loci (liver), interleukin-27 levels, height, metabolite levels, triglyceride levels, serum alkaline phosphatase levels, QT interval, mitochondrial DNA copy number, platelet-to-lymphocyte ratio, total cholesterol levels, breast cancer |

**Abbreviations:** chr (chromosome), pos (position).

**Table S5.** Enriched GO-terms of nearest genes associated with average daily %MVPA.

| **ID** | **Description** | **pvalue** | **ONTOLOGY** | **Count** | **FDR** |
| --- | --- | --- | --- | --- | --- |
| GO:0045974 | regulation of translation, ncRNA-mediated | 2.14e-08 | BP | 37 | 9.21e-05 |
| GO:0090656 | t-circle formation | 6.29e-08 | BP | 4 | 1.35e-04 |
| GO:1904046 | negative regulation of vascular endothelial growth factor production | 4.93e-06 | BP | 21 | 7.08e-03 |
| GO:0098942 | retrograde trans-synaptic signaling by trans-synaptic protein complex | 7.29e-06 | BP | 7 | 7.62e-03 |
| GO:0098969 | neurotransmitter receptor transport to postsynaptic membrane | 8.85e-06 | BP | 75 | 7.62e-03 |
| GO:0007606 | sensory perception of chemical stimulus | 1.00e-04 | BP | 232 | 1.87e-02 |
| GO:0007608 | sensory perception of smell | 1.00e-04 | BP | 213 | 1.87e-02 |
| GO:0009593 | detection of chemical stimulus | 1.00e-04 | BP | 223 | 1.87e-02 |
| GO:0016441 | posttranscriptional gene silencing | 1.00e-04 | BP | 213 | 1.87e-02 |
| GO:0016458 | gene silencing | 1.00e-04 | BP | 218 | 1.87e-02 |
| GO:0031047 | gene silencing by RNA | 1.00e-04 | BP | 215 | 1.87e-02 |
| GO:0035194 | post-transcriptional gene silencing by RNA | 1.00e-04 | BP | 213 | 1.87e-02 |
| GO:0035195 | gene silencing by miRNA | 1.00e-04 | BP | 213 | 1.87e-02 |
| GO:0050906 | detection of stimulus involved in sensory perception | 1.00e-04 | BP | 236 | 1.87e-02 |
| GO:0050907 | detection of chemical stimulus involved in sensory perception | 1.00e-04 | BP | 205 | 1.87e-02 |
| GO:0050911 | detection of chemical stimulus involved in sensory perception of smell | 1.00e-04 | BP | 209 | 1.87e-02 |
| GO:0061550 | cranial ganglion development | 5.52e-05 | BP | 15 | 1.87e-02 |
| GO:1905205 | positive regulation of connective tissue replacement | 4.90e-05 | BP | 5 | 1.87e-02 |
| GO:0033141 | positive regulation of peptidyl-serine phosphorylation of STAT protein | 5.80e-05 | BP | 13 | 1.87e-02 |
| GO:1901725 | regulation of histone deacetylase activity | 6.05e-05 | BP | 11 | 1.87e-02 |
| GO:0015870 | acetylcholine transport | 1.00e-04 | BP | 3 | 1.87e-02 |
| GO:0021924 | cell proliferation in external granule layer | 5.51e-05 | BP | 17 | 1.87e-02 |
| GO:0090370 | negative regulation of cholesterol efflux | 7.39e-05 | BP | 17 | 1.87e-02 |
| GO:0071863 | regulation of cell proliferation in bone marrow | 1.25e-04 | BP | 2 | 2.24e-02 |
| GO:0045002 | double-strand break repair via single-strand annealing | 1.55e-04 | BP | 2 | 2.57e-02 |
| GO:1905167 | positive regulation of lysosomal protein catabolic process | 1.54e-04 | BP | 12 | 2.57e-02 |
| GO:1902645 | tertiary alcohol biosynthetic process | 1.87e-04 | BP | 14 | 2.88e-02 |
| GO:0052695 | cellular glucuronidation | 1.84e-04 | BP | 11 | 2.88e-02 |
| GO:0110011 | regulation of basement membrane organization | 2.09e-04 | BP | 17 | 3.11e-02 |
| GO:1905906 | regulation of amyloid fibril formation | 2.55e-04 | BP | 5 | 3.66e-02 |
| GO:0004984 | olfactory receptor activity | 1.00e-04 | MF | 209 | 2.5e-02 |
| GO:0150100 | RNA binding involved in posttranscriptional gene silencing | 1.00e-04 | MF | 115 | 2.5e-02 |
| GO:1903231 | mRNA binding involved in posttranscriptional gene silencing | 1.00e-04 | MF | 115 | 2.5e-02 |
| GO:0016885 | ligase activity, forming carbon-carbon bonds | 5.59e-05 | MF | 6 | 2.5e-02 |
| GO:0015526 | hexose-phosphate:inorganic phosphate antiporter activity | 5.72e-05 | MF | 6 | 2.5e-02 |
| GO:0052833 | inositol monophosphate 4-phosphatase activity | 1.04e-04 | MF | 15 | 2.5e-02 |
| GO:0000832 | inositol hexakisphosphate 5-kinase activity | 2.43e-04 | MF | 2 | 5.0e-02 |
| GO:0071144 | heteromeric SMAD protein complex | 6.80e-05 | CC | 9 | 2.98e-02 |
| GO:0002139 | stereocilia coupling link | 6.26e-05 | CC | 5 | 2.98e-02 |

Abbreviations: BP, Biological Processes; MF, Molecular Function; CC, Celular Component.

**Table S6**. Enriched GO-terms of nearest genes associated with average daily MET-Hours.

| **ID** | **Description** | **pvalue** | **ONTOLOGY** | **Count** | **FDR** |
| --- | --- | --- | --- | --- | --- |
| GO:0043697 | cell dedifferentiation | 6.95e-07 | BP | 2 | 3.00e-03 |
| GO:0098597 | observational learning | 2.22e-06 | BP | 5 | 3.15e-03 |
| GO:0040033 | negative regulation of translation, ncRNA-mediated | 2.92e-06 | BP | 44 | 3.15e-03 |
| GO:0086027 | AV node cell to bundle of His cell signaling | 1.72e-06 | BP | 10 | 3.15e-03 |
| GO:1905203 | regulation of connective tissue replacement | 6.38e-06 | BP | 5 | 5.51e-03 |
| GO:0021557 | oculomotor nerve development | 8.78e-06 | BP | 31 | 6.32e-03 |
| GO:0021615 | glossopharyngeal nerve morphogenesis | 1.33e-05 | BP | 42 | 8.23e-03 |
| GO:0007606 | sensory perception of chemical stimulus | 1.00e-04 | BP | 200 | 2.06e-02 |
| GO:0007608 | sensory perception of smell | 1.00e-04 | BP | 186 | 2.06e-02 |
| GO:0009593 | detection of chemical stimulus | 1.00e-04 | BP | 192 | 2.06e-02 |
| GO:0016441 | posttranscriptional gene silencing | 1.00e-04 | BP | 241 | 2.06e-02 |
| GO:0016458 | gene silencing | 1.00e-04 | BP | 242 | 2.06e-02 |
| GO:0031047 | gene silencing by RNA | 1.00e-04 | BP | 239 | 2.06e-02 |
| GO:0035194 | post-transcriptional gene silencing by RNA | 1.00e-04 | BP | 241 | 2.06e-02 |
| GO:0035195 | gene silencing by miRNA | 1.00e-04 | BP | 240 | 2.06e-02 |
| GO:0050906 | detection of stimulus involved in sensory perception | 1.00e-04 | BP | 200 | 2.06e-02 |
| GO:0050907 | detection of chemical stimulus involved in sensory perception | 1.00e-04 | BP | 191 | 2.06e-02 |
| GO:0050911 | detection of chemical stimulus involved in sensory perception of smell | 1.00e-04 | BP | 180 | 2.06e-02 |
| GO:0140291 | peptidyl-glutamate ADP-deribosylation | 4.94e-05 | BP | 1 | 2.06e-02 |
| GO:0061589 | calcium activated phosphatidylserine scrambling | 4.41e-05 | BP | 33 | 2.06e-02 |
| GO:1900020 | positive regulation of protein kinase C activity | 8.25e-05 | BP | 3 | 2.06e-02 |
| GO:0071420 | cellular response to histamine | 1.15e-04 | BP | 10 | 2.26e-02 |
| GO:0086017 | Purkinje myocyte action potential | 1.67e-04 | BP | 5 | 3.14e-02 |
| GO:0004984 | olfactory receptor activity | 1E-04 | MF | 180 | 4.81e-02 |
| GO:0150100 | RNA binding involved in posttranscriptional gene silencing | 1E-04 | MF | 110 | 4.81e-02 |
| GO:1903231 | mRNA binding involved in posttranscriptional gene silencing | 1E-04 | MF | 110 | 4.81e-02 |

Abbreviations: BP, Biological Processes; MF, Molecular Function.

**Table S7*.*** The modification effects of BMI on the association between physical activity and methylation levels.

a.

| **cpg** | **trait** | **Model** | **Estimate** | **se** | **P** |
| --- | --- | --- | --- | --- | --- |
| cg27071152 | MVPA | Base model | 8.48e-02 | 8.62e-02 | 3.25e-01 |
| cg27071152 | MVPA | BMI-adjusted | 1.06e-01 | 8.76e-02 | 2.27e-01 |
| cg00484396 | MVPA | Base model | 4.38e-02 | 5.80e-02 | 4.50e-01 |
| cg00484396 | MVPA | BMI-adjusted | 2.65e-02 | 5.89e-02 | 6.53e-01 |
| cg02024705 | MVPA | Base model | -2.69e-02 | 1.97e-02 | 1.73e-01 |
| cg02024705 | MVPA | BMI-adjusted | -2.95e-02 | 2.00e-02 | 1.41e-01 |
| cg21164418 | MVPA | Base model | -2.08e-02 | 2.26e-02 | 3.59e-01 |
| cg21164418 | MVPA | BMI-adjusted | -2.19e-02 | 2.30e-02 | 3.41e-01 |
| cg20630690 | MVPA | Base model | -8.90e-03 | 1.69e-02 | 5.99e-01 |
| cg20630690 | MVPA | BMI-adjusted | -4.81e-03 | 1.72e-02 | 7.80e-01 |
| cg01980521 | MVPA | Base model | -2.62e-02 | 2.22e-02 | 2.39e-01 |
| cg01980521 | MVPA | BMI-adjusted | -2.77e-02 | 2.26e-02 | 2.21e-01 |
| cg12078958 | MVPA | Base model | -4.68e-02 | 2.15e-02 | 2.93e-02 |
| cg12078958 | MVPA | BMI-adjusted | -5.95e-02 | 2.18e-02 | 6.32e-03 |

b.

| **cpg** | **trait** | **Model** | **Estimate** | **se** | **P** |
| --- | --- | --- | --- | --- | --- |
| cg02493524 | MET | Base model | 3.62e-06 | 7.72e-04 | 9.96e-01 |
| cg02493524 | MET | BMI-adjusted | 2.17e-05 | 7.86e-04 | 9.78e-01 |
| cg12325351 | MET | Base model | 1.28e-04 | 2.10e-04 | 5.42e-01 |
| cg12325351 | MET | BMI-adjusted | 1.47e-04 | 2.13e-04 | 4.92e-01 |
| cg21496511 | MET | Base model | -1.05e-04 | 2.43e-04 | 6.67e-01 |
| cg21496511 | MET | BMI-adjusted | -6.66e-05 | 2.48e-04 | 7.88e-01 |
| cg18193094 | MET | Base model | -6.15e-04 | 3.69e-04 | 9.59e-02 |
| cg18193094 | MET | BMI-adjusted | -5.03e-04 | 3.75e-04 | 1.80e-01 |
| cg19237047 | MET | Base model | -2.58e-04 | 2.62e-04 | 3.24e-01 |
| cg19237047 | MET | BMI-adjusted | -2.42e-04 | 2.66e-04 | 3.64e-01 |
| cg23299462 | MET | Base model | -4.17e-06 | 2.58e-04 | 9.87e-01 |
| cg23299462 | MET | BMI-adjusted | 2.28e-05 | 2.63e-04 | 9.31e-01 |
| cg08264338 | MET | Base model | -1.00e-03 | 4.01e-04 | 1.24e-02 |
| cg08264338 | MET | BMI-adjusted | -1.03e-03 | 4.09e-04 | 1.20e-02 |
| cg00056202 | MET | Base model | 8.37e-04 | 5.38e-04 | 1.20e-01 |
| cg00056202 | MET | BMI-adjusted | 8.57e-04 | 5.48e-04 | 1.18e-01 |
| cg17385847 | MET | Base model | 2.87e-04 | 2.46e-04 | 2.44e-01 |
| cg17385847 | MET | BMI-adjusted | 2.18e-04 | 2.51e-04 | 3.86e-01 |
| cg09557462 | MET | Base model | 5.47e-04 | 3.75e-04 | 1.45e-01 |
| cg09557462 | MET | BMI-adjusted | 6.22e-04 | 3.82e-04 | 1.03e-01 |
| cg07093177 | MET | Base model | -2.59e-04 | 4.82e-04 | 5.91e-01 |
| cg07093177 | MET | BMI-adjusted | -5.51e-04 | 4.90e-04 | 2.60e-01 |
| cg15885440 | MET | Base model | -3.92e-04 | 3.66e-04 | 2.85e-01 |
| cg15885440 | MET | BMI-adjusted | -5.96e-04 | 3.73e-04 | 1.10e-01 |

Base model was adjust for age, sex, smoking status, batch and first 10 genetic principal components.

**Figure S1.** Age- and sex-stratified effects of physical activity on GrimAge acceleration.

**a**-**c** Age- and sex-stratified effects on GrimAge acceleration for (**a**) average daily step counts, (**b**) average daily energy expenditure, as expressed in Metabolic-Equivalent Hours (MET-Hours), (**c**) average daily % moderate-to-vigorous intensity activities.

Regression lines were adjusted for education, batch effect, cell proportions, season and smoking status.

**Figure S2.** Scatterplot of association between physical activity and GrimAge acceleration after excluding individuals with high leverage points (highlighted).

Regression lines were adjusted for age, age^2^, sex, education, batch effect, cell proportions, season and smoking status.

**Abbreviations:** Metabolic-Equivalent Hours (MET-Hours).

**Figure S3.** Contributions of cardiovascular variables to factor analysis for mixed data (FAMD) composite components.

**Abbreviations:** Systolic Blood Pressure (SBP), Diastolic Blood Pressure (DBP), High-Density Lipoprotein (HDL), Low-Density Lipoprotein (LDL), Waist-to-Hip Ratio (WHR).

**Figure S4.** Contributions of leukocyte variables to principle component analysis composite components.


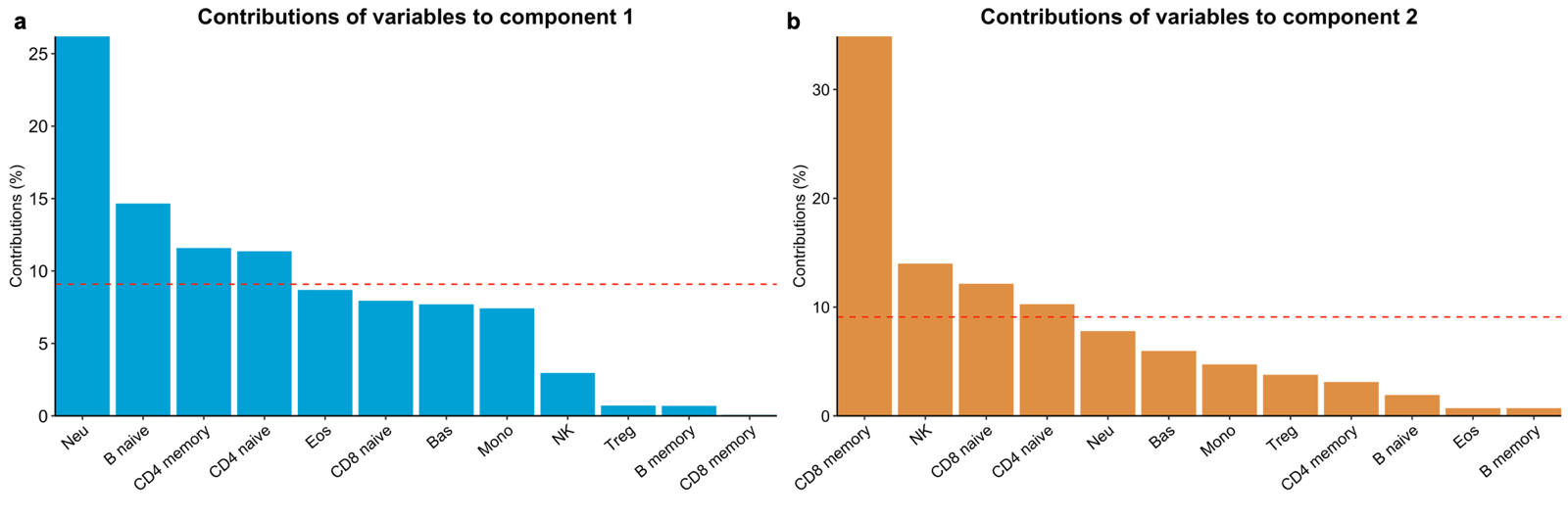


**Abbreviations:** Basophils (Bas), Eosinophils (Eos), Neutrophils (Neu), Monocytes (Mono), Naïve B cells (B naïve), memory B cells (B memory), naïve CD4T cells (CD4 naïve), memory CD4T cells (CD4 memory), regulatory T cells (Treg), naïve CD8T cells (CD8 naïve), memory CD8T cells (CD8 memory) and natural killer cells (NK).

**Figure S5.** Scatterplot of association between physical activity and GrimAge acceleration in individuals without a prior cardiovascular event.

Regression lines were adjusted for age, age^2^, sex, education, batch effect, cell proportions and smoking status.

**Abbreviations:** Metabolic-Equivalent Hours (MET-Hours).

**Figure S6.** Recruitment Flowchart.

**Figure S7**. QQ plot in epigenome-wide association *p*-values for physical activity.

**a**


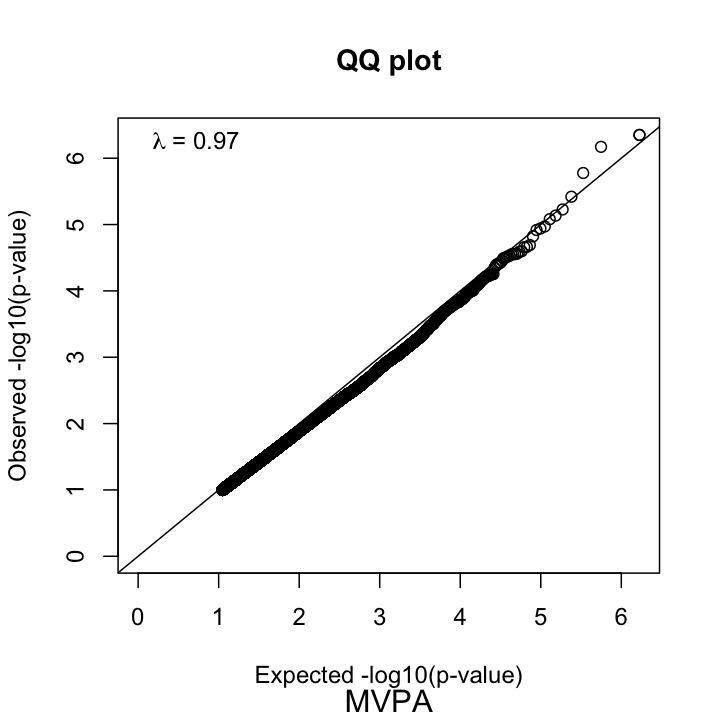


**b**


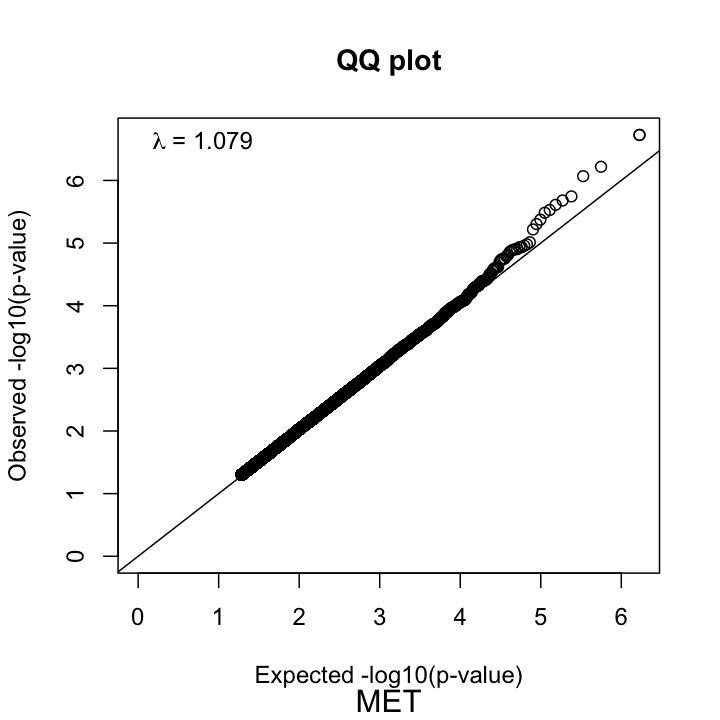


Observed -log10 (p value) are plotted against expected values for (a) % average daily time spent in moderate-to-vigorous activities and (b) average daily energy expenditure in MET-Hours. λ indicates the genomic inflation factor.
